# Moonlighting matrix metalloproteinase substrates: Enhancement of proinflammatory functions of extracellular tyrosyl-tRNA synthetase upon cleavage

**DOI:** 10.1074/jbc.RA119.010486

**Published:** 2019-11-26

**Authors:** Parker G. Jobin, Nestor Solis, Yoan Machado, Peter A. Bell, Simran K. Rai, Nam Hoon Kwon, Sunghoon Kim, Christopher M. Overall, Georgina S. Butler

**Affiliations:** ‡ Department of Biochemistry and Molecular Biology, University of British Columbia, Vancouver, British Columbia V6T 1Z3, Canada; § Centre for Blood Research, University of British Columbia, Vancouver, British Columbia V6T 1Z3, Canada; ¶ Department of Oral Biological and Medical Sciences, Faculty of Dentistry, University of British Columbia, Vancouver, British Columbia V6T 1Z3, Canada; ‖ Graduate Program in Bioinformatics, University of British Columbia, Vancouver, British Columbia V5T 4S6, Canada; ** College of Pharmacy, Seoul National University, 151-742, Seoul, Republic of Korea; ‡‡ Medicinal Bioconvergence Research Center, Seoul National University, 151-742, Seoul, Republic of Korea

**Keywords:** multifunctional protein, aminoacyl tRNA synthetase, proteolysis, matrix metalloproteinase (MMP), inflammation, toll-like receptor (TLR), macrophage, innate immunity, moonlighting proteins

## Abstract

Tyrosyl-tRNA synthetase ligates tyrosine to its cognate tRNA in the cytoplasm, but it can also be secreted through a noncanonical pathway. We found that extracellular tyrosyl-tRNA synthetase (YRS) exhibited proinflammatory activities. In addition to acting as a monocyte/macrophage chemoattractant, YRS initiated signaling through Toll-like receptor 2 (TLR2) resulting in NF-κB activation and release of tumor necrosis factor α (TNFα) and multiple chemokines, including MIP-1α/β, CXCL8 (IL8), and CXCL1 (KC) from THP1 monocyte and peripheral blood mononuclear cell–derived macrophages. Furthermore, YRS up-regulated matrix metalloproteinase (MMP) activity in a TNFα-dependent manner in M0 macrophages. Because MMPs process a variety of intracellular proteins that also exhibit extracellular moonlighting functions, we profiled 10 MMPs for YRS cleavage and identified 55 cleavage sites by amino-terminal oriented mass spectrometry of substrates (ATOMS) positional proteomics and Edman degradation. Stable proteoforms resulted from cleavages near the start of the YRS C-terminal EMAPII domain. All of the MMPs tested cleaved at ADS^386^↓^387^LYV and VSG^405^↓^406^LVQ, generating 43- and 45-kDa fragments. The highest catalytic efficiency for YRS was demonstrated by MMP7, which is highly expressed by monocytes and macrophages, and by neutrophil-specific MMP8. MMP-cleaved YRS enhanced TLR2 signaling, increased TNFα secretion from macrophages, and amplified monocyte/macrophage chemotaxis compared with unprocessed YRS. The cleavage of YRS by MMP8, but not MMP7, was inhibited by tyrosine, a substrate of the YRS aminoacylation reaction. Overall, the proinflammatory activity of YRS is enhanced by MMP cleavage, which we suggest forms a feed-forward mechanism to promote inflammation.

## Introduction

Matrix metalloproteinases (MMPs)^6^ are a family of 23 secreted and membrane-anchored proteases (1). By cleaving a variety of signaling molecules in addition to extracellular matrix components (2), MMPs regulate many different processes, including inflammation (1–7). Notably, the innate immune cell MMPs, neutrophil-specific MMP8 (8) and monocyte/macrophage-lineage MMP7 (9) and MMP12 (14), orchestrate leukocyte chemotaxis during inflammation by activating and inactivating cleavages of chemokines. Indeed, the majority of human CC chemokine ligands (CCL) that regulate monocyte and macrophage chemotaxis and activation are cleaved by these and other MMPs (3, 12, 13), as are all seven human ELR^+^ CXCL neutrophil chemokines, including CXCL8 (IL8). For CCL chemokines, inactivation or generation of antagonists by MMPs are common sequelae, whereas CXCL activation by neutrophil MMP8 is followed later by inactivating cleavages just C-terminal to or within the ELR motif by MMP12 (11), which temporally regulates neutrophil chemoattraction *in vivo* (6, 8, 11, 14). Cytokines such as interferon (IFN) α (10) and IFNγ (7) and complement proteins, including mannose-binding lectin (15), C1q (16), C3, C3a, C3b, and C5a (6), are also MMP substrates *in vivo*. Thus, MMP processing of these bioactive substrates dampens inflammation with MMPs playing beneficial roles essential for terminating inflammatory responses (3, 17).

Several eukaryotic tRNA synthetases have roles within the cell in addition to aminoacylation (18–20) in protein synthesis, and despite lacking a canonical signal sequence, several tRNA synthetases (21–25) have extracellular “moonlighting” functions (26, 27). Very recently, we demonstrated that the proinflammatory activities of moonlighting tryptophanyl-tRNA synthetase (WRS), secreted in response to IFNγ, are lost following MMP cleavage (28). Tyrosyl-tRNA synthetase (YRS) (∼59 kDa, 528 residues), which ligates tyrosine to tRNA(Tyr) in protein translation, is also a moonlighting protein. In addition to an N-terminal Rossmann fold catalytic domain (residues 1–230) and tRNA anticodon recognition domain (residues 231–364), eukaryotic YRS has a C-terminal endothelial monocyte-activating polypeptide II-like (EMAPII) domain (residues 365–528). The EMAPII domain of YRS is 51% identical to the cytokine EMAPII, but is absent from prokaryotic YRS and is not necessary for translation, implying additional functions for the domain (24, 29).

Extracellular YRS has been detected in human plasma (30) and in exosomes (31) and is abundant in releasable platelet granules (25, 30). YRS is secreted from the human histiocytic lymphoma cell line U-937 in culture (32). Truncated proteoforms of YRS, arising from alternative splicing, lack the monocyte chemoattractant EMAPII domain (33). The sequence R^371^VGKIIT^377^ in the EMAPII domain is responsible for chemotaxis (32). The Rossmann fold domain of YRS also displays a neutrophil chemoattractant E^91^LR^93^ motif that was shown to engage the CXCR1 receptor (24). The EMAPII domain released by plasmin and elastase cleavage induces secretion of tumor necrosis factor (TNF)α from macrophages (34). An additional moonlighting function of YRS, which is shared with several tRNA synthetases, is the generation of diadenosine polyphosphates in a side reaction during aminoacylation (35, 36): *P*^1^,*P*^4^-di(adenosine-5′) tetraphosphate (Ap4A) and *P*^1^,*P*^5^-di(adenosine-5′) pentaphosphate (Ap5A). These products also have physiological roles outside the cell. Extracellular Ap4A and Ap5A bind potassium channels on myocardial tissue (37) and inhibit neutrophil apoptosis (38). Thus, YRS may control multiple physiological and disease processes both inside and outside the cell.

To further elucidate the roles of MMPs in physiological and pathological processes that can occur when proteolytic activity is dysregulated, we developed several targeted proteomic technologies known collectively as “degradomics” (39–41). Degradomics identifies protease substrates in both *in vitro* and *in vivo* cell culture systems (41–44), animal models (6, 45), and human tissues (46–49). Amino-terminal oriented mass spectrometry of substrates (ATOMS) is a highly-sensitive targeted approach for identifying mature protein N termini and protease-generated neo N termini in *in vitro* assays (50, 51). Our terminal amine isotopic labeling of substrates (TAILS) degradomics method identifies precise cleavage sites *in vivo* (40, 52), and quantification is enabled by differential isotopic labeling, for example with isobaric tags for relative and absolute quantification (iTRAQ^TM^) (53).

In several TAILS proteomic studies, we previously found that MMPs cleave extracellular YRS. In inflamed murine skin from WT *versus Mmp2*^−/−^ mice, we identified a neo N-terminal peptide, SEPEEVIPSR, resulting from *in vivo* cleavage of YRS at AKN^357^↓S^358^EP (45). In RAW264.7 cell secretomes, MMP12 cleavage of YRS at PRT^401^↓V^402^VS occurred as evidenced from increased levels of the YRS cleavage fragment commencing at the neo N-terminal peptide VVSGLVQFVPKEELQDR (6). Addition of MMP2 to fibroblast secretomes also led to cleavage of YRS at VSG^405^↓L^406^VQ, as shown by identification of the neo N-terminal peptide LVQFVPKEELQDR in MMP2-treated *versus* control cell cultures (20). However, in all these cellular and animal studies, the functional consequences of YRS cleavage by MMPs were not determined. This prompted the present mechanistic follow-up analyses of the cellular responses comparing intact *versus* MMP-cleaved YRS. We confirmed our degradomics evidence that MMPs process YRS *in vivo*; we determined that, unlike WRS (28), MMP cleavage enhances the proinflammatory activity of YRS via TLR2 signaling, and we show that YRS induces MMP activity in a feed-forward mechanism.

## Results

### Proinflammatory effects of YRS on monocytes and macrophages

**Figure 1.**
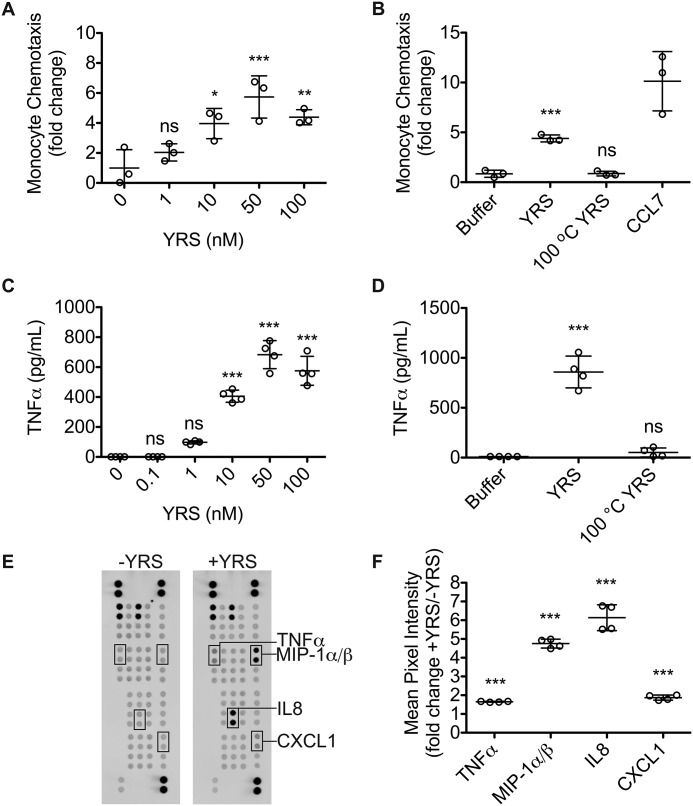
**Increased THP1 monocyte chemotaxis, TNFα secretion, and chemokine secretion induced by YRS.** Transwell chemotaxis assay of THP1 monocytes toward increasing concentrations of recombinant human YRS (*A*) and 50 nm YRS, 50 nm heat-denatured YRS (100 °C YRS) or buffer (*B*). CCL7 (50 nm) was used as a positive control. After 90 min, cells in the lower chamber were counted. Data were plotted as fold change compared with buffer (mean ± S.D., *n* = 3) of *N* = 2 independent experiments for both *A* and *B*. ELISA of TNFα protein levels in THP1 M0 macrophage-conditioned media after treatment for 3 h with increasing concentrations of YRS (*C*) and 50 nm YRS, heat-denatured YRS (100 °C YRS) (*D*), or buffer control (mean ± S.D., *n* = 4) of *N* = 2 independent experiments for each of *C* and *D. E,* cytokine protein levels in the conditioned media of human peripheral blood mononuclear-derived macrophages treated for 3 h ± 50 nm YRS detected by a human cytokine array. The cytokines and chemokines with significant changes in expression ± YRS are *boxed*. See Fig. S2 for identities of cytokine and chemokine spots. *F,* mean pixel intensities from *E* were measured by densitometric analysis and plotted as fold changes + YRS compared with −YRS (mean ± S.D., *n* = 4) of *N* = 2 independent experiments. Statistical significance was determined: against buffer for *A–D* using a one-way ANOVA with Dunnett's multiple comparison post-tests and between ± YRS conditions for *F* using an unpaired two tailed Student's *t* test. ***, *p* < 0.001; *ns*, not significant. *Error bars* represent S.D.

The recruitment of monocytes is a key component of the inflammatory response (54). We expressed and purified human YRS in *Escherichia coli* ClearColi® BL21 (DE3) (55, 56). This strain of *E. coli* produces a lipid variant of lipopolysaccharide (LPS) that does not trigger an endotoxic response in human cells. In an *in vitro* Transwell chemotaxis assay, THP1 monocytes migrated toward YRS with maximal chemotaxis at 50 nm YRS (*N* = 2; Fig. 1*A*). Chemotaxis was abrogated by heat-denaturation of YRS (*N* = 2; Fig. 1*B*). Thus, the tertiary structure of YRS is important for chemotactic signaling, and our recombinant YRS preparation also did not contain any heat-stable chemotaxis agonists.

In inflammation, monocytes and macrophages secrete cytokines and chemokines that amplify the inflammatory response by recruiting additional inflammatory cells (1). THP1 monocyte-derived macrophages (THP1 M0) were differentiated using phorbol 12-myristate 13-acetate (PMA) and shown to constitutively secrete YRS. Secretion was unaffected by treatment with IFNα, IFNβ, IFNγ, or IL4 (Fig. S1, *A* and *B*). Treatment of THP1 M0 macrophages with recombinant YRS for 3 h induced the release of TNFα into the medium (*N* = 2; Fig. 1*C*). This effect was also maximal at 50 nm YRS. TNFα was not released if YRS had been denatured by heating to 100 °C, supporting the requirement for the native tertiary structure of YRS (*N* = 2; Fig. 1*D*). To further explore the stimulation of cytokine and chemokine production by YRS, we prepared human peripheral blood mononuclear cell (PBMC)-derived macrophages. Upon stimulation by 50 nm YRS, the conditioned medium was probed for expression of 36 cytokines and chemokines using protein arrays (Fig. 1 *E* and Fig. S2). Thereby, we confirmed that YRS increased the secretion of TNFα from human PBMC, as well as CCL3 (MIP-1α), CCL4 (MIP-1β), and the ELR^+^ cytokinesCXCL1 (KC) and CXCL8 (IL8) (*N* = 2; Fig. 1, *D* and *F*).

### YRS signals through Toll-like receptor 2 and NF-κB

**Figure 2.**
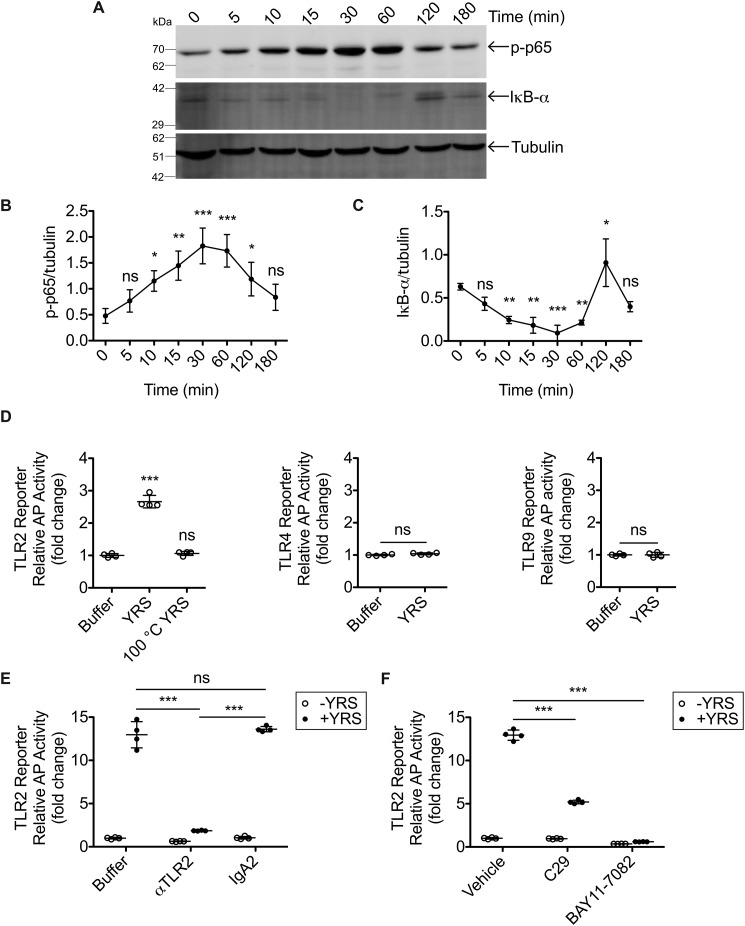
**YRS stimulates NF-κB signaling through TLR2.** *A,* representative immunoblots of phosphorylated (p)-p65 NF-κB and inhibitor of NF-κB (*I*κ*B-*α) following treatment of PMA-differentiated THP1-derived human macrophages with 50 nm recombinant human YRS for the times shown. An immunoblot of α-tubulin is shown as the loading control. Uncropped immunoblots can be found in Fig. S4*C*. Quantification of relative band densities of p-p65 NF-κB (*B*) and IκB-α (*C*) plotted as mean ± S.D. of *N* = 3 independent experiments. *D,* HEK293 cells expressing TLR2, -4, or -9 with a NF-κB alkaline phosphatase (*AP*) reporter system were treated for 18 h with 50 nm recombinant human YRS, heat-denatured YRS (100 °C YRS), or buffer. *E,* TLR2 reporter cells were pre-treated for 1 h with 5 μg/ml TLR2-blocking antibody (α*TLR2*), isotype control IgA2, or buffer prior to treatment ± 50 nm YRS for 18 h. *F,* TLR2 reporter cells were pre-treated for 1 h with 100 μm TLR2 inhibitor C29, 10 μm NF-κB activation inhibitor BAY11-7082 that targets IκB kinase, or vehicle (1% (v/v) DMSO) prior to treatment ± 50 nm YRS for 18 h. The relative activity of alkaline phosphatase was plotted as follows: *D*, fold changes compared with buffer (means ± S.D., *n* = 4) of *N* = 2 independent experiments; TLR9, *N* = 1; *E* and *F*, fold change compared with the buffer −YRS control (means ± S.D., *n* = 4) of *N* = 2 independent experiments. Statistical significance was determined as follows: *B* and *C*, against 0 h using a one-way ANOVA with Dunnett's multiple comparison post-tests; *D*, against buffer using a one-way ANOVA with Dunnett's multiple comparison post-tests for TLR2 and an unpaired two-tailed Student's *t* test for TLR4/9; *E* and *F*, against buffer (or vehicle) and antibody (or inhibitor) treatment in the presence of YRS (+*YRS*) using a two-tailed unpaired Student's *t* test. *, *p* < 0.05; **, *p* < 0.01; ***, *p* < 0.001; *ns*, not significant. *Error bars* represent S.D.

The transcription and release of cytokines such as TNFα from a variety of cells involve the NF-κB signaling pathway that leads to coordination of proinflammatory responses (57). Therefore, we investigated whether the NF-κB pathway is involved in the YRS-mediated stimulation of cytokine and chemokine secretion from macrophages. Treatment of PMA-derived THP1 macrophages with YRS led to phosphorylation of the p65 subunit of NF-κB within 10 min, with maximal levels of phosphorylated (p)-p65 reached at 30 min (*N* = 3; Fig. 2, *A* and *B*). This rapid increase in active p-p65 corresponded to a maximal decrease of the NF-κB inhibitor, IκB-α (*N* = 3; Fig. 2, *A* and *C*), indicating that YRS directly activates the NF-κB–signaling pathway in macrophages.

NF-κB signaling is integral to several pathways, including innate immune responses triggered by pattern recognition receptors such as Toll-like receptors (TLR)2 and TLR4 on macrophages and monocytes (21, 58). Therefore, we screened for YRS activation of TLRs using HEK293 TLR NF-κB reporter cell lines: YRS (50 nm) stimulated NF-κB reporter signaling through TLR2 but not TLR4 or the cytosolic DNA receptor TLR9 (*N* = 2; Fig. 2*D*). TLR2 signaling was abolished by denaturation of YRS at 100 °C, consistent with the effect of heat denaturation leading to loss of monocyte chemotaxis (Fig. 1*B*) and TNFα release from macrophages (Fig. 1*D*). Moreover, a TLR2-neutralizing antibody and TLR2 pathway inhibitors blocked NF-κB activation in the HEK293 TLR2 reporter cells (*N* = 2; Fig. 2, *E* and *F*).

**Figure 3.**
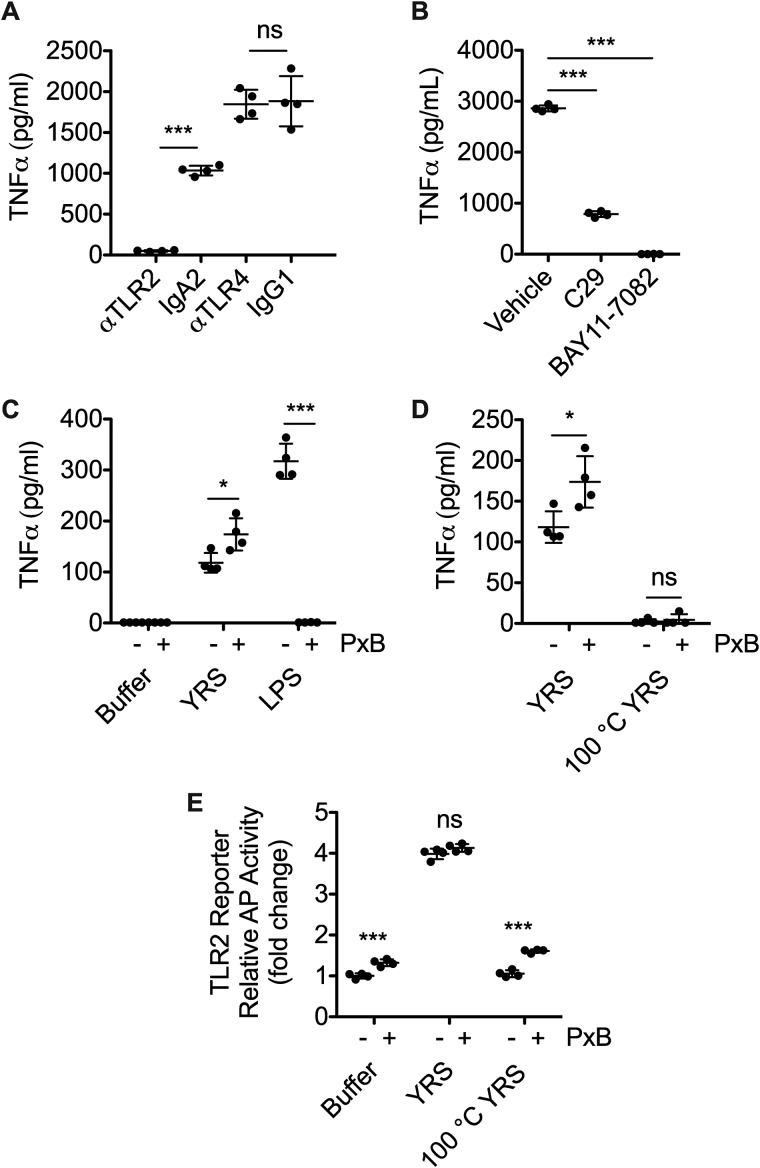
**Inhibition of TLR2 signaling reduces YRS-mediated TNFα release from THP1 macrophages.** TNFα released to the conditioned medium of PMA-differentiated THP1-derived macrophages in response to treatment with 50 nm recombinant human YRS for 3 h after pre-treatment for 1 h with 5 μg/ml TLR2- or TLR4-blocking antibodies (αTLR2, αTLR4) or isotype controls (IgA2, for αTLR2; IgG1, for αTLR4) (mean ± S.D., *n* = 4) of *N* = 3 independent experiments (*A*), or TLR2 inhibitor C29 (100 μm), IκB kinase inhibitor BAY11-7082 (10 μm), or vehicle (1% (*v*/*v*) DMSO) (mean ± S.D., *n* = 4) of *N* = 2 independent experiments (*B*). TNFα measured by ELISA in the conditioned media of PMA-differentiated THP1-derived macrophages treated for 3 h ± 10 μg/ml polymyxin B (*PxB*) with 50 nm YRS, 100 ng/ml LPS, or buffer (*C*) or 50 nm heat-denatured YRS (100 °C YRS) (*D*). Data were plotted as means ± S.D. (*n* = 4) of *N* = 3 independent experiments. *E,* HEK293 cells expressing TLR2 with an NF-κB alkaline phosphatase (*AP*) reporter system were treated for 18 h with 50 nm recombinant human YRS, heat-denatured YRS (100 °C YRS) or buffer ± 10 μg/ml polymyxin B. The relative activity of alkaline phosphatase was plotted as fold changes compared with buffer, polymyxin B (means ± S.D., *n* = 4) of *N* = 2 independent experiments. Statistical significance was determined as follows. *A*, between each isotype control and antibody using a two-tailed unpaired Student's *t* test; *B*, against vehicle using a one-way ANOVA with Dunnett's multiple comparison post-tests; *C–E,* between the −*PxB* and +*PxB* conditions using a two-tailed unpaired Student's *t* test. *, *p* < 0.05; ***, *p* < 0.001; *ns,* not significant. *Error bars* represent S.D.

We used these antibodies and inhibitors to demonstrate that YRS initiates NF-κB signaling through TLR2 in macrophages. The TLR2-blocking antibody (5 μg/ml) reduced TNFα production in THP1-derived macrophages by 95% compared with its IgA2-isotype control (*N* = 3; Fig. 3*A*), whereas a TLR4-blocking antibody, which was effective in inhibiting LPS initiation of TLR4 signaling in reporter cells (Fig. S3), failed to reduce TNFα compared with the IgG1 isotype control in THP1 M0. TNFα production by THP1 M0 in response to YRS was also reduced by 74% by C29 (100 μm), a small molecule inhibitor of TLR2 signaling that blocks myeloid differentiation primary response gene 88 recruitment to TLR2 (59), and was inhibited completely by BAY11-7082 (10 μm), a potent NF-κB nuclear translocation inhibitor that targets IκB kinase (*N* = 2; Fig. 3*B*) (60, 61). Thus, blocking either the extracellular domain of TLR2 with an αTLR2 antibody or interfering with intracellular components of the NF-κB–signaling pathway using chemical inhibitors disrupted YRS-induced TNFα secretion, demonstrating that TLR2/NF-κB mediates this proinflammatory response to YRS in human macrophages.

As recombinant proteins expressed in *E. coli* can be contaminated with LPS, a heat-stable activator of TLR signaling, the recombinant YRS used in all of the experiments was expressed in *E. coli* ClearColi®, which produces a variant LPS that does not trigger TLR signaling. Additional measures were also taken to eliminate any LPS contamination: inclusion of the LPS scavenger Triton X-114 during YRS purification and polymyxin B–agarose extraction of any LPS from the purified recombinant YRS before use. In addition, we included polymyxin B in all cell-culture experiments to remove any contaminating environmental endotoxin. To validate the efficacy of our strategy in eliminating any TLR-stimulating endotoxins in our experiments, we assessed TNFα release from THP1-derived macrophages. Polymyxin B (10 μg/ml) blocked TNFα release from M0 macrophages in response to 100 ng/ml LPS, but it did not reduce TNFα release stimulated by 50 nm YRS (*N* = 3; Fig. 3*C*). In the absence of polymyxin B, heat denaturation of YRS eliminated TNFα release confirming the absence of the heat-stable endotoxin (*N* = 3; Fig. 3*D*). Confirmatory results were obtained using HEK293 TLR2 reporter cells; polymyxin B did not inhibit the activity of YRS, but heat denaturation of YRS abolished TLR2 stimulation (*N* = 2, Fig. 3*E*).

### TNFα induces MMPs that cleave YRS

**Figure 4.**
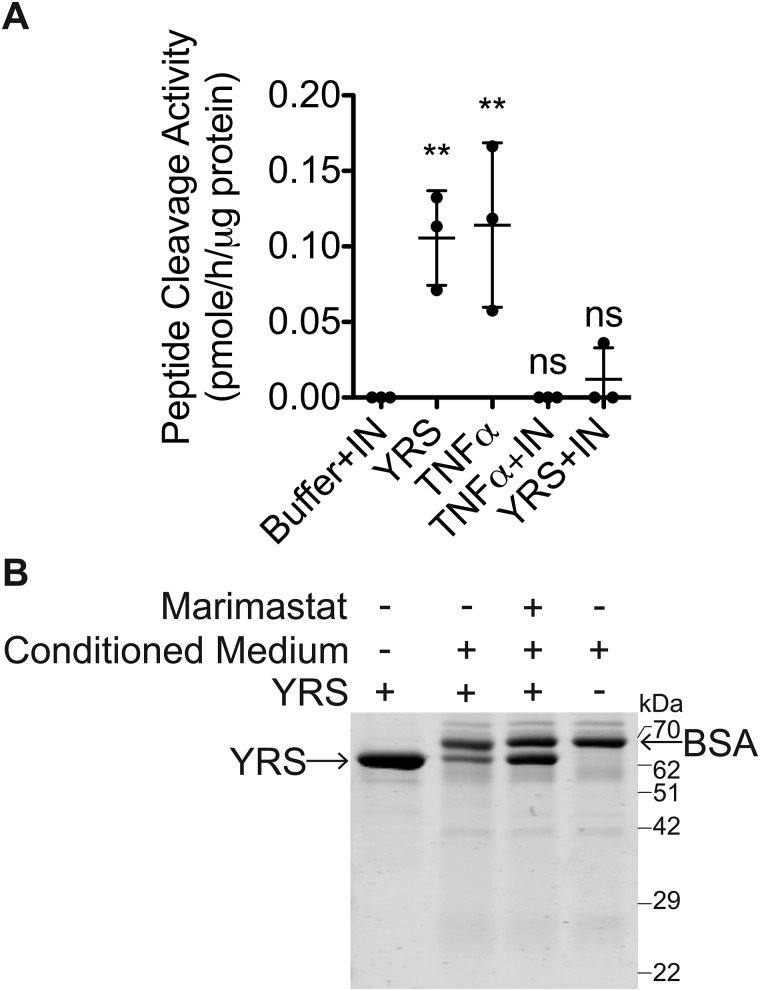
**YRS increases macrophage-secreted MMP activity in a TNFα-dependent manner.** *A,* PMA-differentiated THP1-derived macrophages were treated for 24 h with 50 nm recombinant human YRS, or 40 ng/ml TNFα, or buffer, with or without a mAb inhibitor of TNFα (100 ng/ml inflixamab, *IN*). MMPs in the concentrated (20×) conditioned media were activated with 1 mm APMA, and cleavage of a quenched fluorescence peptide substrate Mca-Pro-Leu-Gly-Leu-Dpa-Ala-Arg-NH_2_ was measured (mean ± S.D., *n* = 3) in *N* = 3 independent experiments. Statistical significance was determined against “buffer + inflixamab” using a one-way ANOVA with Dunnett's multiple comparison post-test. **, *p* < 0.01; ns, not significant. *Error bars* represent S.D. *B,* to induce MMP expression, PMA-differentiated THP1-derived macrophages were treated for 24 h with 40 ng/ml TNFα. MMPs in the concentrated (20×) conditioned media were activated with 1 mm APMA for 20 min at 22 °C prior to addition of YRS (∼62 kDa) ± MMP inhibitor (10 μm marimastat), at a protein ratio of 5:1 medium protein/YRS, for 18 h at 37 °C. YRS-containing conditioned medium samples were resolved by 10% SDS-PAGE and stained with Coomassie Brilliant Blue G-250. The ∼66-kDa band is BSA in the concentrated conditioned medium. A representative gel of *N* = 3 independent experiments is shown. The uncropped gel is shown in Fig. S5*A*.

TNFα increases MMP expression (62). Because YRS stimulated TNFα secretion from macrophages, we assessed whether YRS could induce MMP expression via TNFα. THP1-derived macrophages were treated with YRS (50 nm) or TNFα (50 ng/ml) with or without the TNFα inhibitory monoclonal antibody inflixamab (100 ng/ml). MMP activity in the conditioned medium was measured by cleavage of a quenched fluorescent MMP peptide substrate (*N* = 3; Fig. 4*A*). Treatment of the macrophages with YRS markedly increased MMP activity in the medium to a level comparable with that induced by TNFα. Inflixamab negated not only the TNFα-stimulated increase in MMP activity, but also that stimulated by YRS, suggesting that YRS indirectly induced MMP secretion via TNFα.

Our previous degradomics studies identified YRS as a substrate of MMP2 and MMP12 (6, 20). Hence, we explored whether the MMPs induced by YRS treatment of macrophages could cleave YRS. Conditioned medium was harvested from TNFα-treated M0 macrophages, and secreted MMPs were activated by 1 mm *p*-aminophenylmercuric acetate (APMA) (64) prior to incubation with recombinant human YRS. Incubation led to a strong reduction in the intensity of the 62-kDa YRS band visualized by Coomassie Brilliant Blue staining after SDS-PAGE (*N* = 3; Fig. 4*B*). This cleavage was abrogated by marimastat (10 μm), a broad-spectrum metalloproteinase inhibitor (65). Thus, metalloproteinase activity in the conditioned media cleaved YRS suggesting a possible feedback mechanism where YRS drives MMP expression through TNFα, and these proteases then cleave YRS.

### YRS is cleaved by MMPs

**Figure 5.**
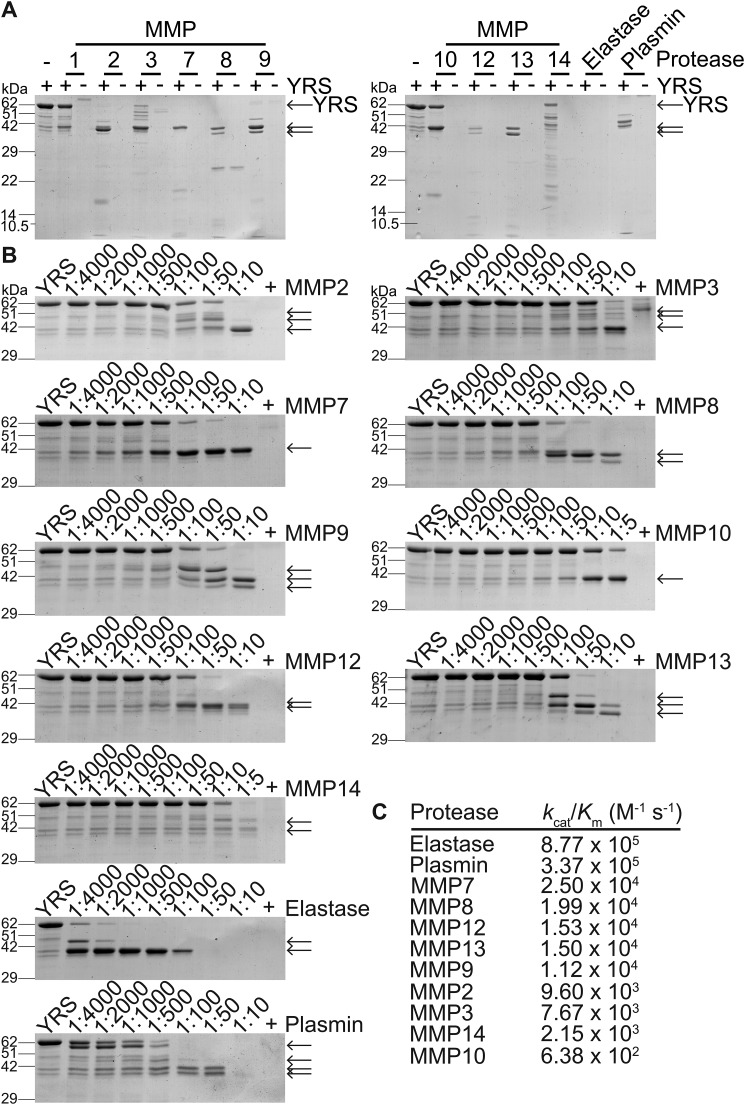
**Coomassie Brilliant Blue-stained SDS-PAGE analysis of YRS cleavage by recombinant MMPs *in vitro*.** *A,* recombinant human YRS (∼62 kDa) was incubated for 18 h at 37 °C with recombinant MMPs, neutrophil elastase, or plasmin (1:10 molar ratio protease/YRS), *N* = 2 independent experiments. *B,* YRS was incubated for 18 h at 37 °C at the protease/YRS molar ratios shown. Uncropped gels are shown in Fig. S5, *B* and *C*. Controls (+) are the highest concentration of protease without YRS. Cleavage products were analyzed by 12% (*A*) or 10% (*B*) SDS-PAGE with Coomassie Brilliant Blue G-250 staining. Cleavage products are indicated by *arrows. C, k*_cat_/*K_m_* values for cleavage of YRS.

To elucidate which MMPs can cleave YRS, we conducted *in vitro* cleavage assays with ten MMPs that we expressed, including MMP2 and MMP12 that we had shown in cell secretomes and *in vivo* cleave YRS (6, 40). We compared cleavage by the serine proteases neutrophil elastase and plasmin, as both cleave YRS at Ser^366^↓Arg^367^, separating the Rossmann fold and EMAPII domains (24, 34). Before incubation, the activity of each MMP was confirmed using quenched fluorescent peptide cleavage assays. At a 1:10 molar ratio MMP/YRS, MMP2, MMP7, MMP8, MMP9, MMP12, and MMP13 processed YRS completely from 62 kDa to a major ∼41-kDa form (*N* = 2; Fig. 5*A*). MMP8, MMP9, and MMP13 produced a doublet, similar to that produced by plasmin. Partial cleavage of YRS was found by MMP1, MMP3, and MMP10, whereas MMP14 generated multiple cleavage fragments indicative of degradation.

We compared cleavage efficiencies and potential differences in specificity by incubating YRS with each MMP at a range of molar ratios and calculated kinetic parameters using densitometry (Fig. 5, *B* and *C*). The four MMPs with the highest cleavage efficiency for YRS, namely MMP7, MMP8, MMP12, and MMP13, generated a stable fragment migrating at ∼41 kDa at a 1:100 molar ratio MMP/YRS. At higher molar ratios of MMP7, the ∼41-kDa fragment was stable, whereas higher ratios of the serine proteases, neutrophil elastase and plasmin, resulted in complete degradation of YRS (Fig. 5*B*). The efficiency of the MMPs varied considerably with a 40-fold difference in *k*_cat_/*K_m_* for YRS cleavage between MMP7, the most efficient MMP with a *k*_cat_/*K_m_* 2.50 × 10^4^ m^−1^ s^−1^, and the least efficient, MMP10.

**Figure 6.**
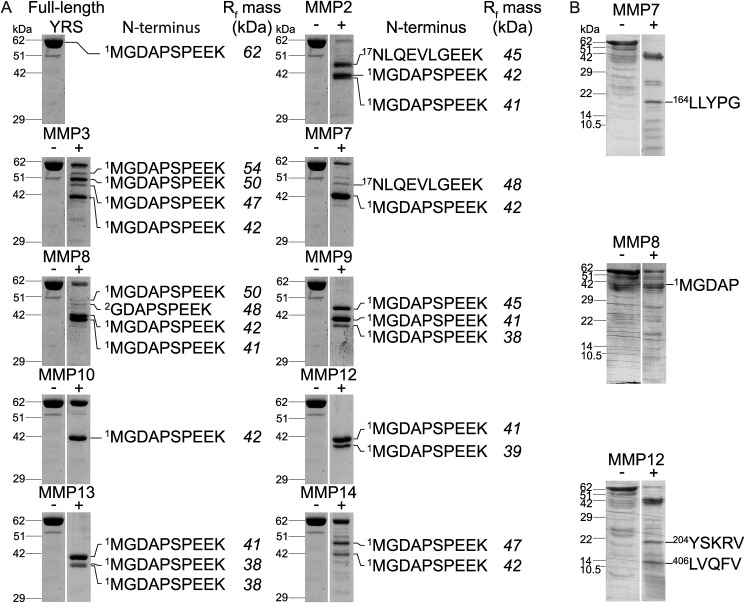
**MMP-cleaved YRS protein bands sequenced by LC-MS/MS and Edman degradation.** *A,* LC-MS/MS was used to identify the most N-terminal peptide of MMP-cleaved YRS bands. After MMP digestion of 2 μm recombinant human YRS for 18 h at 37 °C (MMP/YRS molar ratios used were guided by results shown in Fig. 5*B*), cleavage products or the full-length ∼62 kDa YRS (*top left*) were resolved by 10% SDS-PAGE and stained with Coomassie Brilliant Blue G-250. Bands were excised, digested with trypsin, and analyzed by LC-MS/MS. N-terminal peptides identified from YRS cleavage products after trypsin digestion are shown. Molecular masses shown in *italics* were estimated from *R_f_* values. The YRS negative control lane was duplicated here between MMP2, MMP3, MMP7, MMP8, and MMP9 and again between MMP10, MMP12, MMP13, and MMP14 according to the grouping of digests on the same gels for analysis as shown on the uncropped full gels presented in Fig. S5*D*. *B,* YRS was incubated for 18 h at 37 °C ± MMP7, MMP8, or MMP12 at 1:10 MMP/YRS molar ratios. Cleavage products were resolved by 16.5% SDS-PAGE, transferred to PVDF membrane, stained with Coomassie Brilliant Blue G-250, and subjected to Edman degradation. N-terminal sequences are shown and the original stained blots, from which bands were excised for Edman degradation, are shown in full in Fig. S6.

To identify the cleavage sites of MMPs in YRS, we excised YRS cleavage fragments from SDS-polyacrylamide gels and performed in-gel trypsin digests followed by LC-MS/MS (Fig. 6*A*, Supplementary Table S1–S25). The predominant 41-kDa fragment and additional major cleavage products commenced either at the original N terminus of YRS or for MMP2 and MMP7 at Arg^16^↓Asn^17^. We conclude that the cleavage of YRS from 62 to 41 kDa was mediated by C-terminal cleavages. Indeed, Edman degradation sequencing confirmed that the 41-kDa band generated by MMP8 contained the N terminus of YRS, whereas N-terminal sequencing of lower molecular weight cleavage products generated by MMP7 and MMP12 identified cleavage at Gly^163^↓Leu^164^ and Gly^203^↓Tyr^204^, respectively, within the YRS catalytic domain, and a more C-terminal cleavage by MMP12 at Gly^405^↓Leu^406^ (Fig. 6*B*).

**Figure 7.**
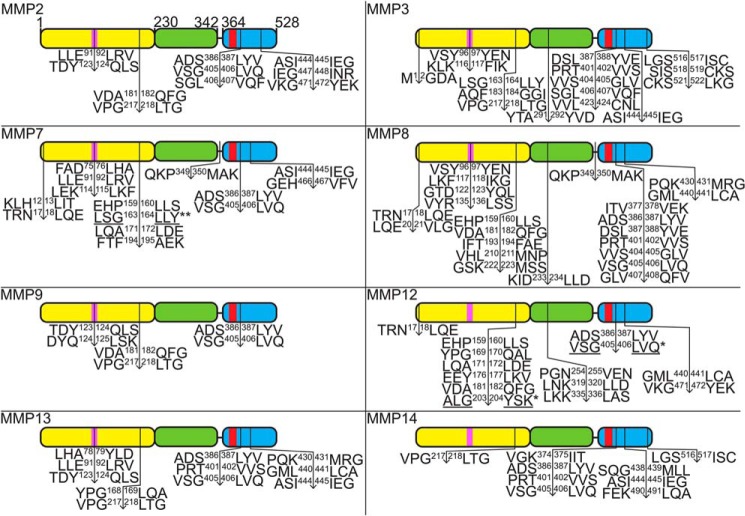
**Identification of MMP cleavage sites in YRS.** Major and minor MMP cleavage sites in recombinant human YRS were determined by ATOMS N-terminal positional proteomics or by Edman degradation of proteins blotted to PVDF membranes as shown in Fig. 6*B*. Cleavage sites identified by ATOMS (↓), Edman degradation (*, *underlined*), or by both (**) are shown. Note, ATOMS is a highly-sensitive technique that identifies cleavage sites, but does not determine relative cleavage rates. Thus, several ATOMS sites were not observed by SDS-PAGE due to the low abundance of their cleavage fragments. *Yellow*, Rossmann fold catalytic domain; *green*, anticodon recognition domain; *blue*, endothelial monocyte-activating polypeptide II-like (EMAPII) domain; *pink*, E^91^LR^93^ tripeptide motif; *red*, R^371^VGKIIT^377^ heptapeptide motif.

**Figure 8.**
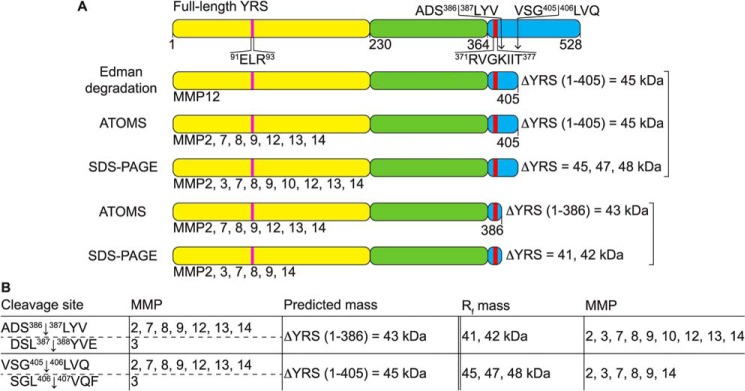
**Major stable YRS proteoforms generated by MMP cleavage of YRS.** *A,* MMP cleavage sites in YRS were determined by N-terminal sequencing by Edman degradation of proteins shown in Fig. 6*B* or by ATOMS N-terminal positional proteomics shown in Fig. 7. Schematic diagrams of YRS with the MMP cleavage sites leading to predicted N-terminal fragments of YRS (ΔYRS) are shown. Fragments predicted from cleavage sites are aligned by molecular mass with fragments observed on SDS-polyacrylamide gels in Fig. 6*A*. Molecular masses of predicted fragments were determined using the ExPASy tool (https://web.expasy.org/compute_pi/), and the molecular mass of YRS fragments observed by SDS-PAGE were determined from relative migration distances compared with molecular mass standards (*R_f_*). MMPs generating each fragment are indicated. *Yellow,* Rossmann fold catalytic domain; *green*, anticodon recognition domain; *blue*, endothelial monocyte-activating polypeptide II-like (EMAPII) domain; *pink*, E^91^LR^93^ tripeptide motif; *red,* R^371^VGKIIT^377^ heptapeptide motif. *B,* table of cleavage sites common to all MMPs identified by ATOMS and Edman sequencing. Predicted mass, the calculated molecular mass of the ΔYRS(1–383) and ΔYRS(1–405) cleavage products. *R_f_*, apparent molecular masses of the major cleavage fragments resolved by electrophoresis.

Proteomic N-terminal sequencing by ATOMS revealed MMP cleavage hot spots in the Rossmann fold domain of YRS between residues ∼160 and 220 and between residues ∼90 and 130 for some, but not all 10 MMPs that were profiled (Fig. 7, Supplementary Table S26–S41). However, all 10 MMPs cleaved close to the beginning of the C-terminal EMAPII-like domain at VSG^405^↓^406^LVQ, as well as at ADS^386^↓^387^LYV, or at DSL^387^↓^388^YVE (MMP3) (Figs. 7 and 8*B*). N-terminal fragments generated by these cleavages, YRS(1–386) and YRS(1–405), have predicted molecular masses of 43 and 45 kDa, respectively (Fig. 8), that are comparable with the major YRS cleavage products identified by SDS-PAGE (Figs. 6*A* and 8). Thus, the N-terminal fragment of YRS remaining after release of the EMAPII domain by MMPs contains both the ELR motif and the chemotactic heptatpeptide (R^371^VGKIIT^377^) that is now positioned 10 residues away from the neo C terminus of the major cleavage fragment.

### MMP cleavage enhances YRS-induced proinflammatory responses

**Figure 9.**
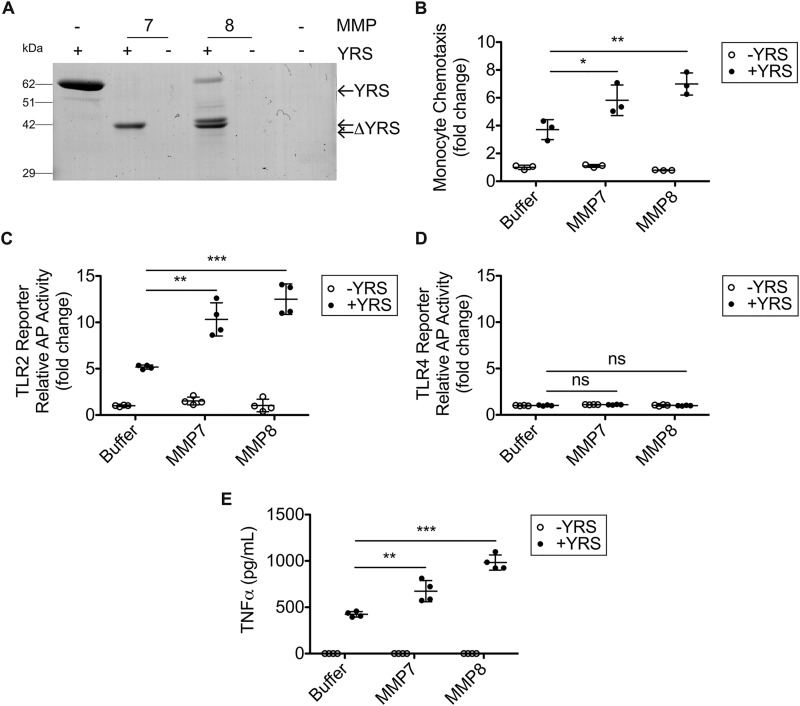
**MMP7 and MMP8 cleavage of YRS increases monocyte chemotaxis, TLR2 signaling, NF-κB activation, and TNFα release by macrophages.** *A,* cleavage of recombinant human YRS (∼62 kDa) by MMP7 and MMP8 (1:10 protease/YRS molar ratio) for 18 h at 37 °C visualized by Coomassie Brilliant Blue G-250-stained 10% SDS-PAGE. ΔYRS-cleavage products. The uncropped gel is presented in Fig. S5*D*. *B,* transwell chemotaxis assay (90 min) of THP1 monocytes migrating toward 50 nm YRS, YRS cleaved by MMP7 or MMP8, MMPs alone or buffer. Data are presented as fold change compared with buffer alone (mean ± S.D., *n* = 3) of *N* = 3 independent experiments. HEK293 cells co-expressing TLR2 (*C*) or TLR4 (*D*) and a NF-κB alkaline phosphatase (*AP*) reporter system were treated for 18 h with 50 nm YRS, YRS cleaved by MMP7 or MMP8, MMPs alone or buffer. The relative activity of alkaline phosphatase is plotted as fold change for TLR compared with buffer alone (mean ± S.D., *n* = 4) of *N* = 2 independent experiments for both *C* and *D. E,* ELISA measurement of TNFα released to the conditioned media of PMA-differentiated THP1-derived macrophages treated for 3 h with 50 nm intact YRS, MMP7, or MMP8-cleaved YRS or MMPs alone or buffer (plotted as mean ± S.D., *n* = 4) of *N* = 3 independent experiments. Statistical significance was determined between YRS treated with buffer and YRS cleaved by MMP7 and MMP8 using a two-tailed unpaired Student's *t* test. *, *p* < 0.05; **, *p* < 0.01; ***, *p* < 0.001; *ns,* not significant. *Error bars* represent S.D.

The 3D structure of YRS shows that the ELR chemotactic motif in the Rossmann fold catalytic domain and the heptapeptide sequence in the EMAPII domain are masked in folded full-length YRS (66). We hypothesized that MMP cleavage exposes these sequences upon removal of the EMAPII domain to increase biological activities such as chemotaxis. We focused on MMP7 and MMP8, because both are secreted by innate immune cells and showed the highest cleavage efficiency for YRS (Fig. 5*C*). In accordance with the hypothesis, cleavage of YRS by MMP7 and MMP8 (Fig. 9*A*) increased THP1 monocyte chemotaxis in the Transwell assay 1.6- and 1.9-fold, respectively, compared with full-length YRS (50 nm) (*N* = 3; Fig. 9*B*). Activation of NF-κB in the HEK293 TLR2 reporter system, but not in the TLR4 reporter system, was also enhanced 2.0- and 2.4-fold by MMP7 and MMP8 cleavage, respectively, compared with intact YRS (50 nm) (*N* = 2; Fig. 9, *C* and *D*). Additionally, TNFα release from THP1-derived macrophages increased 1.6- and 2.3-fold after treatment with MMP7- and MMP8-cleaved YRS, respectively, compared with intact YRS (50 nm) (*N* = 3; Fig. 9*E*). Thus, MMP7 and MMP8 cleavage amplifies the proinflammatory activity of YRS.

### Tyrosine inhibits MMP8-mediated cleavage of YRS

**Figure 10.**
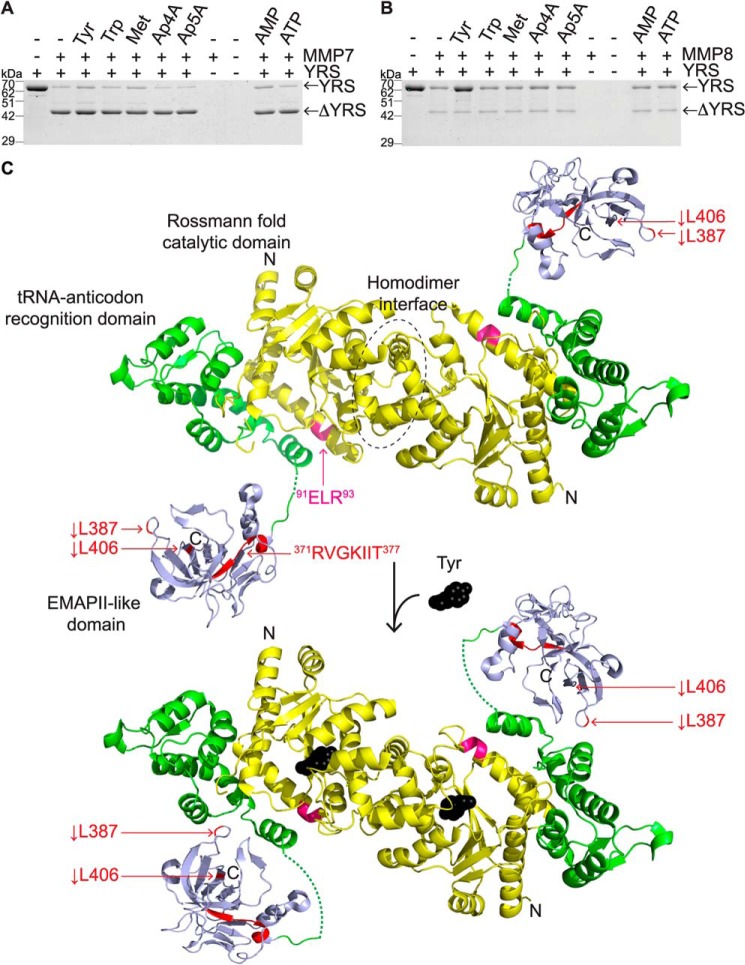
**MMP8 cleavage of YRS is inhibited by Tyr *in vitro*.** Recombinant human YRS (∼62 kDa) was pre-incubated with 0.5 mm Tyr, Trp, Met, Ap4A, Ap5A, AMP, ATP, or buffer as shown for 30 min at room temperature. Recombinant human MMP7 (*A*) or MMP8 (*B*) was added (1:100 MMP/YRS molar ratio) and incubated for 18 h at 37 °C. Cleavage products (ΔYRS) were analyzed by 10% SDS-PAGE, *N* = 2 independent experiments. Cleavage products were visualized by Coomassie Brilliant Blue G-250 staining and are indicated by *arrows. C,* structural model of apo-YRS (*upper*) and with bound Tyr (*lower*) showing major sites of MMP cleavage. The 3D structures of N-terminal YRS (residues 1–342) homodimers without (*above*, PDB 1N3L) and with (*below*, PDB 4QBT) Tyr bound. The Rossmann fold catalytic domains (*yellow*) and the tRNA-anticodon recognition domains (*green*) essential for aminoacylation, were modeled with the 3D structure of the C-terminal EMAPII-like domain (*blue*) of YRS (PDB 1NTG) to represent a full-length YRS molecule. The sequences of E^91^LR^93^ and R^371^VGKIIT^377^, important for the cytokine activity of YRS, are shown in *pink* and *red,* respectively. MMP8 cleavage sites *L387* and *L406* are in *red*.

After demonstrating that YRS is a substrate of MMPs, we tested whether substrates and products of two YRS-mediated reactions, aminoacylation and diadenosine polyphosphate synthesis (35, 36), could influence MMP cleavage of YRS. Reaction substrates tyrosine and ATP and products AMP, Ap4A, and Ap5A were preincubated with YRS before incubation with MMP7 or MMP8 (Fig. 10). Tryptophan and methionine were incubated as negative controls. Although MMP7 cleavage of YRS was unaffected by these substrates and products (*N* = 2, Fig. 10*A*), the presence of tyrosine reduced proteolytic cleavage of YRS by MMP8 (*N* = 2, Fig. 10*B*). Thus, the previously reported structural change in the N-terminal domains of YRS that occurs upon binding tyrosine (67) reduces MMP8 cleavage. We localized the major MMP7 and MMP8 cleavage sites on the 3D structure of YRS that we modeled from two partial YRS structures, the N-terminal Rossmann fold catalytic and anticodon recognition domains (residues 1–342), which occur as a dimer (29, 67), and the isolated EMAPII-like C-terminal domain (Fig. 10*C*) (66).

## Discussion

MMP cleavage of YRS was identified in murine skin inflammation from our earlier TAILS proteomic analyses of inflamed *versus* normal skin in WT *versus Mmp2*^−/−^ mice (45), murine *Mmp2*^−/−^ fibroblasts (40), and murine *Mmp12*^−/−^ macrophage secretomes (6) with added MMP2 or MMP12. Here, we have expanded the repertoire of proteases that cleave YRS in the extracellular milieu and characterized the biological consequences of MMP cleavage of extracellular moonlighting YRS. MMP cleavage of YRS deleted a large C-terminal peptide that leaves the R^371^VGKIIT^377^-chemoattractive peptide just 10 amino acid residues from the neo C terminus (Fig. 10*C*). We showed that MMP cleavage enhanced NF-κB activation through TLR2, monocyte chemotaxis, and elevated secretion of TNFα, MIP-1α/β, CXCL8 (IL8), CXCL1 (KC) and MMPs from macrophages.

Recently, we demonstrated that the proinflammatory activities of WRS are lost following MMP cleavage (28). Conversely, here we show that MMP7 and MMP8 cleavages within the C-terminal EMAPII domain at Ser^386^↓Leu^387^ and Gly^405^↓Leu^406^ enhance the proinflammatory activities of YRS, highlighting differences between different secreted amino-tRNA synthetases. These cleavage sites are supported by our previous TAILS degradomics analyses that identified MMP-dependent cleavage of YRS at AKN^357^↓S^358^EP (MMP2) (45), VSG^405^↓L^406^VQ (MMP2) (40), and PRT^401^↓V^402^VS (MMP12) (6). MMP regulation of the cytokine activity of YRS has previously been reported only for the *Drosophila melanogaster* homologue of MMP2; YRS secreted from apoptotic *D. melanogaster* hemocytes acted as a chemoattractant of hemocytes, as did MMP2-generated YRS fragments (an N-terminal 41-kDa fragment and a C-terminal 17-kDa fragment), both promoting clearance of the dying cells (68). Unfortunately, the cleavage site within *D. melanogaster* YRS was not determined.

The ELR motif and the chemoattractant heptapeptide of YRS occur at the interface of the Rossmann fold catalytic and the C-terminal EMAPII domains (66) (*see* Fig. 10*C*). The serine proteases elastase and plasmin cleave prior to the chemotactic heptapeptide in the disordered linker between the anticodon recognition and the EMAPII domains: Neutrophil elastase cleaves YRS at Ala^355^↓Lys^356^ and Ser^366^↓Arg^367^, whereas plasmin cleaves at Lys^352^↓Gly^353^ and Lys^356^↓Gln^357^ (34). Previously, neutrophil elastase was also shown to produce YRS(1–364) from recombinant YRS lacking the R^371^VGKIIT^377^ sequence, but which retained leukocyte chemoattractant activity through the ELR motif (69). Cleavage at or near Asp^343^↓Pro^344^ by serine proteases releases the N-terminal domain containing the ELR, and the EMAPII domain with the R^371^VGKIIT^377^-sequence at its cleaved N terminus. Each of these fragments had a distinct activity (24). Serine protease cleavage (24, 34), a gain–of–function mutation (Y341A), and alternative splicing (25) are proposed to induce conformational changes that are responsible for unmasking YRS cytokine activity.

Cleavages of YRS within the EMAPII domain 10 residues C-terminal to the chemotactic heptapeptide R^371^VGKIIT^377^, including at ADS^386^↓^387^LYV and VSG^405^↓^405^LVQ, were common among the majority of the 10 MMPs we tested. In this way, MMP-mediated cleavage deletion of a large C-terminal peptide from position 387 to 528 generates N-terminal fragments of 43 and 45 kDa that contain *both* the ELR motif, which binds CXCR1, the IL8 chemokine receptor on neutrophils, and the chemotactic heptapeptide R^371^VGKIIT^377^ that chemoattracts macrophages and stimulates TNFα secretion (Fig. 8*A*) (32). Thus, MMP cleavage of YRS generates major products that fundamentally differ from the serine protease cleavage products and likely increases YRS signaling by unmasking these sequences, in particular by exposing R^371^VGKIIT^377^ at the new C terminus.

This bioactive YRS fragment displaying both motifs resembles that generated by caspase-7 cleavage of the aminoacyl tRNA synthetase-interacting protein p43 at E^390^EVD^393^ (70). That the MMP N-terminal cleavage fragment exhibits both biological activities is indicated from analysis of an alternative spliced truncation of intracellular YRS(1–380), identified by transcript analyses in leukocytes and spleen (33), which displays both the ELR and the RVGKIIT motifs that are active together in this proteoform of YRS (25). Thus, full-length YRS, like many chemokines, requires proteolytic processing to unmask bioactive sequence motifs for full cytokine activity in contrast to WRS cleavage by MMPs that inactivates its proinflammatory activities (28). Using our highly-sensitive ATOMS proteomics approach, we identified a minor cleavage event by MMP2, -7, and -13 within the bioactive E^91^↓LR motif, that has previously been demonstrated to be an inactivating cleavage in chemokines (11, 71).

We determined that YRS not only triggered TNFα release, but also secretion of multiple chemokines, including MIP-1α/β, CXCL8 (IL8), and CXCL1 (KC), from both THP1-derived and PBMC primary macrophages. Furthermore, YRS activated NF-κB signaling through TLR2, and MMP cleavage enhanced this signaling, presumably due to conformational change induced by truncation that exposed reactive motifs. Like intact YRS, MMP-cleaved YRS failed to activate TLR4 signaling in reporter cells, showing specificity between these receptors for pro-inflammatory activity of YRS and its truncations. Interestingly, native YRS was previously found to bind directly to, but not to stimulate, TLR4, whereas YRS with a conformation-changing Y341A mutation activated TLR2 and TLR4 signaling in human and murine leukocytes (25). Thus, cleavage, alternate splicing or mutation of YRS is required to fully activate TLR2 signaling, with mutation leading to an enhanced repertoire of TLR responsiveness not present in the WT protein.

As the EMAPII domain was shown to trigger TNFα secretion (24), which we found was TLR2-dependent, it is likely that TLR2 recognizes this domain of YRS. Our novel finding of TLR2 signaling may explain the bioactivity observed in full-length YRS that was not reported previously for studies employing elastase and plasmin. Those studies focused on CXCR1 stimulation by the ELR motif in the N-terminal Rossmann fold domain, and other receptor interactions were either not explored or not reported. Future research should address which elements of the EMAPII domain of YRS are necessary to engage and activate TLR signaling.

Substrates and products of YRS-mediated aminoacylation or diadenosine polyphosphate synthesis reactions, including ATP, AMP, Ap4A, and Ap5A, did not influence the MMP-mediated cleavage of YRS. However, tyrosine, a nonessential amino acid derived from phenylalanine that normally exists in the blood at ∼50–100 μm (72), inhibited the cleavage of YRS by MMP8, but not MMP7. Tyrosine binding induces a conformational change in the C-terminal domain of YRS that inhibits the interaction with poly(ADP-ribose) polymerase 1 (PARP-1) in the cell, decreasing the activation of PARP-1 stress signaling mediated by YRS (67). Similarly, tryptophan binding to WRS in the nucleus causes a shift in its homodimer structure from an “open” to a “closed” configuration where the N-terminal domain of WRS folds over the active site (73). When this occurs, WRS within the nucleus can no longer act as a bridging molecule in a complex with DNA-dependent protein kinase and PARP-1 in p53 signaling. Tyrosine might induce a similar structural change in YRS, burying the MMP8 cleavage site and preventing cleavage or affecting MMP binding and thus cleavage. A substrate-binding interaction that involves the MMP8 hemopexin domain would explain why MMP8, but not MMP7, which lacks a hemopexin domain, is affected by tyrosine.

Dysregulated phenylalanine and tyrosine metabolism (hyperphenylalaninemia) occurs in patients with chronic inflammatory conditions (74–76). Whether this involves the modulation of the proinflammatory activity of YRS through modulation of MMP8 cleavage is unclear. Thus tyrosine appears to regulate the proinflammatory activity of YRS through modulation of MMP8 cleavage. It would be interesting to test whether MMP-cleaved fragments of YRS have altered activities in areas other than inflammation, such as synthesis of adenosine polyphosphates, which have their own moonlighting activities (77–79).

**Figure 11.**
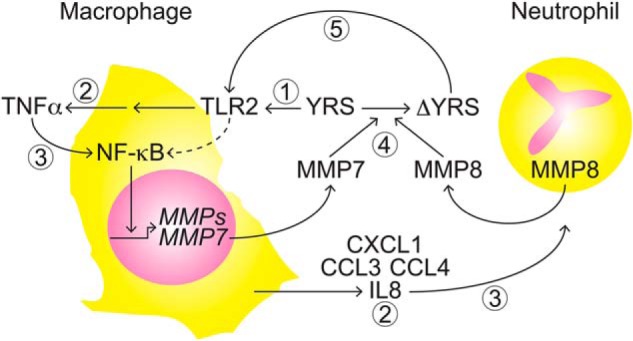
**Feed-forward model of the YRS-MMP temporal relationship in the induction and potentiation of proinflammatory pathways.** *1*) Extracellular YRS activation of TLR2 signaling; *2*) triggering TNFα and chemokine release; *3*) chemokines recruit neutrophils and TNFα and NF-κB activation up-regulate MMP expression; *4*) macrophage MMP7 and neutrophil MMP8 processing of YRS generates truncated proteoforms (ΔYRS). *5*) ΔYRS drives inflammation by enhanced activation of TLR2 signaling in a feed-forward mechanism.

TNFα induces MMP expression and activity from macrophages through signaling pathways, including NF-κB (62). Through the use of a TNFα-blocking antibody, we showed that the release of TNFα by YRS promoted an increase in macrophage metalloproteinase activity. We propose a feed-forward mechanism where proteoforms of YRS generated by MMP cleavage are involved in the recruitment of monocytes that differentiate into macrophages at the site of inflammation (Fig. 11). As macrophages release MMPs that cleave and enhance the inflammatory properties of YRS, we suggest this would accentuate the inflammatory response. MMP8 is a neutrophil-specific protease that not only activates IL8 (8), but also inactivates the elastase inhibitor α_1_-antitrypsin to facilitate highly-efficient activating cleavage of IL8 by elastase *in vivo* (80) in a feed-forward mechanism of IL8 activation for neutrophil chemotaxis (8, 80). As MMPs cleave and inactivate several serine protease inhibitors, including α_2_-antiplasmin (81), to relieve serine protease inhibition at different phases of the inflammatory process, cleavage by elastase and plasmin could further regulate net YRS activity. Interestingly, we found that neutrophil elastase and plasmin completely degraded YRS at higher molar ratios, whereas MMPs generated stable products. We predict that MMP cleavage would inactivate the plasmin- or elastase-generated EMAPII domain by release of a neo N-terminal peptide from Ser^366^ to Ser^386^ containing the chemotactic RVGKIIT heptapeptide to dampen inflammation in a delayed negative feedback loop. In Fig. 4*B*, the stable 41-kDa fragment of YRS generated *in vitro* by purified MMPs was absent after incubation with MMP-containing macrophage-conditioned medium suggesting that other proteases in this relatively complex milieu can further degrade MMP-generated proteoforms of YRS. In view of the multiple MMPs that cleave YRS, forestalling incisive use of *Mmp* knockout mice to further dissect their role in mouse models, the challenge now is to assess the mechanism of YRS regulation by MMPs in the context of healthy and diseased states *in vivo* to assess the depth of this proteolytic tuning of the highly-regulated inflammatory response.

## Experimental procedures

### YRS expression and purification

Human YRS(1–528) with a C-terminal His-tag in pET28a was expressed in ClearColi® BL21(DE3) (Lucigen) in Lennox Broth under kanamycin (20 μg/ml) selection. Harvested cell pellets were resuspended in ice-cold column buffer (20 mm KH_2_PO_4_, 500 mm NaCl, 10 mm imidazole, 2 mm β-mercaptoethanol, pH 7.8) with protease inhibitors (1 mm 4-(2-aminoethyl) benzenesulfonyl fluoride, 1 mm EDTA, 10 μm E-64, 10 μm leupeptin, and 1 μm pepstatin A). Clarified cell lysates were treated with Triton X-114, as described previously (82). Treated lysates were applied to a nickel-nitrilotriacetic acid Perfect Pro (Qiagen) column that was washed with column buffer at pH 7.8, pH 6, and then pH 5.2, and bound protein was eluted with a 25–250 mm imidazole gradient in column buffer. Protein-containing fractions were pooled and dialyzed against PBS, 15% glycerol. Pooled fractions were passed through a polymyxin B–agarose column (Millipore-Sigma) before aliquoting and storage at −80 °C. Protein concentration was determined by bicinchoninic acid assay (Thermo Fisher Scientific).

### Cell culture

The human monocytic cell line THP1 (ATCC no. TIB-202) was cultured in RPMI growth medium: RPMI 1640, 4.5 g/liter glucose, 10% (v/v) cosmic calf serum (CCS), 50 μg/ml streptomycin, and 50 units/ml penicillin. THP1 M0 macrophages were generated by differentiation of THP1 monocytes (1 × 10^6^ cells/ml) with 100 ng/ml PMA, as described previously (7). Experiments were performed using PMA-differentiated THP1 M0 macrophages synchronized for 24 h in serum-free medium containing 10 μg/ml polymyxin B. For NF-κB pathway analyses, THP1 M0 cells were treated with 50 nm YRS for various times. Cells were washed with PBS and lysed with 50 mm Tris-HCl, 150 mm NaCl, 10 mm EDTA, 0.2% (w/v) Zwittergent, pH 8, containing a protease and phosphatase inhibitor mixture (Biotool). Centrifuged cell lysates were assessed using immunoblotting as described below. For TNFα experiments, THP1 M0 cells were treated for 3 h with 50 nm YRS, heat-denatured YRS (boiled for 5 min), *E. coli* 0111:B4 lipopolysaccharide (LPS) (Millipore-Sigma), or PBS. TNFα in conditioned media was measured using a human TNFα ELISA kit (Duoset® ELISA, R&D Systems). 5 μg/ml TLR2-blocking antibody (maba2-htlr2, InvivoGen), TLR4-blocking antibody (mabg-htlr4, InvivoGen), IgA2 isotype control antibody (maba2-ctrl, InvivoGen), IgG1 isotype control antibody (mabgl-ctrlm, InvivoGen), 1% (v/v) DMSO (vehicle), 100 μm C29 (MedChemExpress), 10 μm BAY11-7082 (Selleckchem), or a combination thereof were added 1 h prior to treatment with 50 nm YRS for 3 h for TLR inhibitor experiments.

Human PBMCs were obtained from Ficoll-Pacque^TM^ PLUS (GE Healthcare) separated blood samples. Healthy volunteers donated blood according to a University of British Columbia Clinical Research Ethics Board approved protocol (no. H06-00047). All donors provided informed consent, and research was conducted in accordance with the Declaration of Helsinki. Monocytes were isolated from the human PBMCs using an EasySep^TM^ human monocyte isolation kit (Stemcell) according to the manufacturer's instructions. Monocytes (2 × 10^5^/ml) were differentiated into primary macrophages (M0) by culturing in ImmunoCult^TM^-SF macrophage medium containing 50 ng/ml monocyte–colony-stimulating factor (both Stemcell) for 4 days. For cytokine and chemokine array analysis, serum-starved PBMC-derived M0 were treated for 3 h in serum-free growth medium, 10 μg/ml polymyxin B with 50 nm YRS or PBS. Conditioned media, cleared by centrifugation, were assayed using a Human Cytokine Array® (no. ARY005B, R&D Systems) as per the manufacturer's instructions. Membranes were blocked with Odyssey Blocking Buffer (LI-COR) for 20 min, before a 1-h incubation at 25 °C with streptavidin conjugated to Alexa Fluor® 680 (Thermo Fisher Scientific). A LI-COR Odyssey IR imager was used for imaging. Spots were quantified by densitometry using ImageJ software (National Institutes of Health).

### Monocyte chemotaxis

YRS, heat-denatured YRS, CCL7 (all 50 nm), or PBS in RPMI 1640 medium, 20 mm HEPES, 0.1% bovine serum albumin (BSA), 10 μg/ml polymyxin B (chemotaxis buffer) was added to the lower wells of chemotaxis chambers (Neuroprobe). THP1 monocytes, synchronized by serum starvation, were resuspended in chemotaxis buffer (1 × 10^6^ cells/ml), and 2 × 10^5^ cells were placed in the top wells separated from the lower chamber by a 5-μm membrane. After incubation for 90 min at 37 °C, cells were harvested from the lower well and quantified using CyQUANT® (Thermo Fisher Scientific). CCL7 was synthesized as described previously (12).

### TLR reporter assays

Human embryonic kidney 293 (HEK)-Blue^TM^ cells co-expressing the NF-κB reporter system and receptors TLR2, TLR4, TLR9, or receptor-null counterparts (Null1 and Null2) (all InvivoGen) were cultured in DMEM growth medium (DMEM, 4.5 g/liter glucose, 10% (v/v) CCS, 50 μg/ml streptomycin, and 50 units/ml penicillin) with selective antibiotics. Cell maintenance and assays were conducted according to manufacturer's instructions. For reporter assays, 96-well plates were seeded with TLR2 and Null1, TLR4 and Null2, or TLR9 and Null1 HEK-Blue cells and treated with 50 nm YRS, heat-denatured YRS, or PBS with 10 μg/ml polymyxin B for 18 h. NF-κB activation was assayed using conditioned medium and QUANTI-Blue^TM^ detection medium (InvivoGen). For TLR inhibitor experiments, cells were incubated with antibodies and inhibitors for 1 h before treatment with 50 nm YRS for 18 h as for THP1-derived macrophages above. For TLR4-blocking antibody testing with LPS, cells were incubated with antibodies as above before treatment with 100 ng/ml LPS or PBS in polymyxin B-free medium.

### YRS secretion assays

PMA-differentiated THP1 M0 macrophages were transferred to serum-free media with 100 ng/ml human IFNα (Cedarlane), 100 ng/ml IFNβ, 20 ng/ml IFNγ, or 40 ng/ml IL4 (all Peprotech). Clarified conditioned media were harvested at the times shown, and protease inhibitor mixture (Biotool) and 1 mm EDTA were added. Cells were washed with PBS and lysed in buffer containing protease inhibitor mixture and 1 mm EDTA as above.

Proteins in conditioned media were precipitated by 15% (v/v) TCA, and the resulting protein pellets were washed with 100% acetone. Air-dried pellets were re-solubilized by boiling for 5 min in 4× SDS-PAGE loading buffer (0.5 m Tris, 8 m urea, 8% (w/v) SDS, pH 6.8), and then diluted 4-fold in water. Protein concentrations were determined by absorbance measurements at *A*_280 nm_.

### Immunoblotting

Reduced samples were separated using 10% SDS-PAGE and transferred to PVDF membranes (Immobilon-FL, Millipore-Sigma). Membranes were blocked with Odyssey Blocking Buffer for 20 min, after which primary antibodies (at concentrations recommended by the manufacturers) in PBS with 5% BSA, 0.05% Tween 20 were added and incubated overnight at 4 °C. Primary antibodies were as follows: affinity-purified polyclonal antibody for the N terminus of YRS (no. A305-064A, Bethyl Laboratories); monoclonal antibodies phospho-NF-κB p65 (Ser-536; no. 3033), and total IκB-α (no. 4814) (both Cell Signaling Technology); α-tubulin (no. sc-53646, Santa Cruz Biotechnology). After washing the membranes in PBS with 0.05% Tween 20, secondary antibodies were incubated (at concentrations recommended by manufacturers) in PBS with 1% BSA, 0.05% Tween 20 for 1 h at 25 °C. Secondary antibodies were as follows: goat anti-mouse IgG conjugated to IRDye 800CW (LI-COR) and goat anti-rabbit IgG conjugated to Alexa Fluor® 680 (Thermo Fisher Scientific). Immunoblots were washed and analyzed using a LI-COR Odyssey IR imager, and immunoreactive bands were quantified by densitometry using ImageJ software (National Institutes of Health).

### MMP expression, purification, and activity assays

Recombinant human MMP1, MMP2, MMP3, MMP7, MMP8, MMP9, MMP13, and MMP14 without the transmembrane helix and murine MMP10 and MMP12 were produced as described previously (83). A quenched fluorescence synthetic peptide substrate cleavage assay using Mca-Pro-Leu-Gly-Leu-Dpa-Ala-Arg-NH_2_ (R&D Systems) was used to confirm MMP activation, as described previously (83, 84). THP1-derived M0 macrophages were cultured in phenol red-free RPMI 1640 serum-free growth medium with 10 μg/ml polymyxin B (Millipore-Sigma) incubated with 50 nm YRS, heat-denatured YRS, 40 ng/ml TNFα, or PBS ± 100 ng/ml inflixamab (no. NBP2-52655, Novus Biologicals). Conditioned medium, clarified by centrifugation, was concentrated 20× using Ultra-4 Centrifugal Filter Units (3.5 kDa cut-off) (Amicon). MMP activity in 40 μg of conditioned medium protein was quantified by quenched fluorescence peptide cleavage activity as above.

### YRS cleavage assays

YRS was incubated for 18 h at 37 °C in HEPES buffer: 50 mm HEPES, 150 mm NaCl, 5 mm CaCl_2_, pH 7.2, at various protease/YRS molar ratios. Human neutrophil elastase (Millipore-Sigma) and plasmin (Biovision) were reconstituted in HEPES buffer. All pro-MMPs were activated in HEPES buffer containing 1 mm APMA for 20 min at 22 °C. APMA was removed by dialysis against HEPES buffer at 4 °C. The efficiency of YRS cleavage was assessed by densitometry using ImageJ software (National Institutes of Health), and the data were fitted to Equation 1, as described previously (85):

(Eq. 1) $$k_{\text{cat}}/K_{m}=\left(\ln2\right)/\left[E\right]t_{1/2}$$

To test the effect of YRS substrates and products on MMP7 and MMP8 cleavage of YRS, tyrosine, tryptophan, methionine, AMP, ATP, Ap4A, and Ap5A (0.5 mm) were incubated with YRS at 22 °C for 30 min. MMP7 and MMP8 (1:100 MMP/YRS molar ratio) were added as above.

Clarified conditioned media from THP1 M0 cells cultured in serum-free growth medium with 40 μg/ml TNFα for 24 h were concentrated 20× as above. Concentrated media were treated with 1 mm APMA for 20 min at 25 °C, and APMA was removed as before. YRS was added at a 1:5 protein ratio of YRS/medium total protein and incubated for 18 h at 37 °C. YRS cleavage products were visualized by SDS-PAGE analysis, Coomassie Brilliant Blue-250 staining, and Western blotting.

### MMP cleavage of YRS for sequence analysis

YRS (1 μg) was digested ± MMPs for 18 h at 37 °C using molar ratios MMP/YRS:MMP2, 1:10; MMP3, 1:10; MMP7, 1:50; MMP8, 1:50; MMP9, 1:50; MMP10, 1:5; MMP12, 1:50; MMP13, 1:50; and MMP14, 1:10. Reactions were diluted into SDS-PAGE sample buffer (4×: 0.5 m Tris, 8% (w/v) SDS, pH 6.8, 20% (v/v) β-mercaptoethanol) and boiled for 5 min before resolving by 10% SDS-PAGE.

### LC-MS/MS analysis of YRS cleavage products

MMP-digested YRS bands were stained with Coomassie Brilliant Blue G-250 and excised from the gels, destained with methanol, and lyophilized. Gel slices were rehydrated in 15 μl of MS-grade trypsin (12 ng/μl in 50 mm ammonium bicarbonate) for 45 min at 4 °C. After pulse-centrifugation, buffer was removed, and the gel plugs were resuspended in 50 mm ammonium bicarbonate and incubated at 37 °C for 18 h. After centrifugation as above, polyacrylamide plugs were discarded, and supernatants were desalted using StageTips (86).

Samples were analyzed by LC-MS/MS as described previously (28). Briefly, samples were analyzed using an Easy nLC-1000 (Thermo Fisher Scientific) online-coupled to a UHR Q-TOF Impact II mass spectrometer (Bruker Daltronics) with a CaptiveSpray nanoBooster ionization interface. Sample peptides (1 μg) were automatically loaded onto a 75-μm × 400-mm analytical column (ReproSil-Pur C18 1.8-μm stationary phase resin; Dr. Maisch GmbH) maintained at a column temperature of 50 °C at 800 bars using buffer A (0.1% formic acid) and 8 μl of flush volume for injection. Peptide elution was controlled by a 125-min gradient of buffer B (99.9% acetonitrile and 0.1% formic acid) generated with the nLC, 200 nl/min from 2 to 24% buffer B, 90 min; increased to 30%, 10 min; increased to 95%, 5 min; finally held at 95%, 15 min. An alternative separation strategy of a 60 min gradient with 20 min separation was used with comparable washing parameters. Peptides were ionized using electrospray ionization (2.2 kV). MS analysis was done in positive ion polarity with precursor ion mass tolerance set from 150 *m/z* to 1750 *m/z*. The top 17 ions per scan were selected for collision-induced dissociation using a selection window that varied with *m/z* ranging from 2.5 atomic mass units (300 *m/z*) up to 4 atomic mass units (1300 *m/z*) and at a MS/MS scan rate of three spectra/s. Collision energy was calculated automatically depending on the charge state of the parent ions, and precursor ions were then excluded from further collision-induced dissociation for 30 s after acquisition of two spectra.

Database searching was performed by converting Bruker .d files to .mgf using DataAnalysis version 4.3 (Bruker Daltonics) and searched with the Mascot version 2.5.1 search algorithm (MatrixScience). The mass tolerances of the searches were 25 ppm for MS1 and 0.08 Da for MS2. Enzyme specificity was semi-tryptic. The number of missed cleavages permitted was 1. Variable modifications included methionine oxidation, propionamide cysteine, glutamine and glutamic acid N-terminal ammonia loss, and N-terminal cyclization. Parameters were searched against the reverse concatenated *Homo sapiens* Uniprot database (the UP_human_Canon_Oct2014; date Oct. 1; 69,085 entry sequences). Search result files were imported to Scaffold version 4.8.7 (Proteome Software) and searched using X!Tandem version 2017.2.1.4 (87). Search results were filtered for 1% false discovery rate at both peptide and protein levels. The N-terminal peptides of MMP-cleaved YRS proteoforms excised from the gels were determined from the identified peptides. The MS data has been deposited with the ProteomeXchange Consortium (88) via the PRIDE partner repository (89) with the dataset identifier PXD013197.

### MMP cleavage site determination by ATOMS

YRS cleavage sites were determined by Edman degradation (performed by the Tufts University Core Facility), as described previously (50, 51), and by ATOMS (50, 51). Briefly, YRS (100 μg) was incubated ±MMP (at molar ratios detailed above) at 37 °C for 18 h. Proteins were denatured in 4 m guanidine-HCl, and cysteines were reduced using 5 mm DTT for 1 h at 37 °C and alkylated using 15 mm iodoacetamide for 15 min at room temperature in the dark. Excess iodoacetamide was quenched with 15 mm DTT for 30 min at 37 °C. Lysine and N-terminal amine groups in MMP-digested samples were labeled using 40 mm heavy formaldehyde (C^13^D_2_O), and in samples without MMPs using light formaldehyde (CH_2_O), both in the presence of 20 mm sodium cyanoborohydride at 37 °C for 18 h. Excess formaldehyde was quenched with 50 mm ammonium bicarbonate at 37 °C for 2 h. Samples were mixed, split equally between two tubes, and digested with either 1 μg/ml MS-grade trypsin (Thermo Fisher Scientific) (protease specificity C-terminal cleavage of lysine and arginine residues) or 1 μg/ml GluC (*Staphylococcus aureus* protease V8, Worthington) (protease specificity C-terminal cleavage of glutamic acid and aspartic acid residues) for 16 h at 37 °C. Samples were desalted using StageTips (86) and analyzed by LC-MS/MS as described above, and cleavage sites were identified as described previously (50, 51). Cleavage sites beginning with a hydrophilic or a charged residue at the P1′ position (except for cysteine and glutamine) were not further considered as they did not match the major substrate preferences derived from >4,000 MMP cleavage sites (83). The ATOMS MS data have been deposited as above with the dataset identifier PXD013366.

### Statistics

GraphPad Prism version 5.0b software was used to perform all statistical testing as detailed in the figure legends.

### Molecular modeling

The homodimer 3D structure models of N-terminal YRS (residues 1–342) without (PDB 1N3L) (29) and with (PDB 4QBT) (67) bound tyrosine were compared. Because a full-length human YRS 3D structure was not available, the 3D structure of C-terminal EMAPII-like domain (PDB 1NTG) (66) was used to simulate a full-length YRS molecule. The figure was generated with PyMOL software.

## Author contributions

P. G. J., N. S., and Y. M. data curation; P. G. J., N. S., Y. M., P. A. B., and S. K. R. formal analysis; P. G. J. investigation; P. G. J. methodology; P. G. J. writing-original draft; N. S., Y. M., P. A. B., C. M. O., and G. S. B. writing-review and editing; S. K. R. visualization; N. H. K., S. K., and C. M. O. resources; C. M. O. and G. S. B. conceptualization; C. M. O. and G. S. B. supervision; C. M. O. and G. S. B. funding acquisition; C. M. O. and G. S. B. project administration.

## Supplementary Material

Supporting Information

## Footnotes

6 The abbreviations used are:

MMP: matrix metalloproteinase
Ap4A: *P*^1^,*P*^4^-di(adenosine-5′) tetraphosphate
Ap5A: *P*^1^,*P*^5^-di(adenosine-5′) pentaphosphate
APMA: *p*-aminophenylmercuric acetate
ATOMS: amino-terminal oriented mass spectrometry of substrates
CCL: CC chemokine ligand
CCS: cosmic calf serum
EMAPII: endothelial monocyte-activating polypeptide II
IFN: interferon
IL: interleukin
iTRAQ: isobaric tags for relative and absolute quantification
LPS: lipopolysaccharide
PARP-1: poly(ADP-ribose) polymerase 1
PBMC: peripheral blood mononuclear cell
PMA: phorbol 12-myristate 13-acetate
RPMI: Roswell Park Memorial Institute
TAILS: terminal amine isotopic labeling of substrates
TLR: toll-like receptor
TNF: tumor necrosis factor
WRS: tryptophanyl-tRNA synthetase
YRS: tyrosyl-tRNA synthetase
PDB: Protein Data Bank
ELISA: enzyme-linked immunosorbent assay
ANOVA: analysis of variance
PVDF: polyvinylidene difluoride.
