# Supplementary material for: Moonlighting matrix metalloproteinase substrates: Enhancement of proinflammatory functions of extracellular tyrosyl-tRNA synthetase upon cleavage
Source: J Biol Chem. 2019 Nov 26;295(8):2186–202. doi: 10.1074/jbc.RA119.010486 (PMC7039567; doi:10.1074/jbc.RA119.010486)
Supplement: Supporting Information [file supp_295_8_2186__index.html]

Moonlighting matrix metalloproteinase substrates: Enhancement of proinflammatory functions of extracellular tyrosyl-tRNA synthetase upon cleavage — MMPs enhance proinflammatory YRS activity — Moonlighting matrix metalloproteinase substrates: Enhancement of proinflammatory functions of extracellular tyrosyl-tRNA synthetase upon cleavage — MMPs enhance proinflammatory YRS activity — Supporting Information 

# Moonlighting matrix metalloproteinase substrates: Enhancement of proinflammatory functions of extracellular tyrosyl-tRNA synthetase upon cleavage

## Supporting Information

- Supporting Information (to be published online) - Supplementary information file containing supplementary figures for online publication.
- Supporting Information (to be published online) - Supplementary information file containing supplementary tables for online publication.
