# Supplementary material for: Moonlighting matrix metalloproteinase substrates: Enhancement of proinflammatory functions of extracellular tyrosyl-tRNA synthetase upon cleavage
Source: J Biol Chem. 2019 Nov 26;295(8):2186–202. doi: 10.1074/jbc.RA119.010486 (PMC7039567; doi:10.1074/jbc.RA119.010486)
Supplement: Supporting Information [file supp_RA119.010486_154947_1_supp_423033_q0bf6m.pdf]

Moonlighting matrix metalloproteinase substrates: Enhancement of proinflammatory functions of extracellular tyrosyl-tRNA synthetase upon cleavage

Parker G. Jobin[1,2], Nestor Solis[2,3], Yoan Machado[2,3], Peter A. Bell[2,3], Simran K. Rai[2,4], Nam Hoon Kwon[5,6], Sunghoon Kim[4,6], Christopher M. Overall[1-3#*], and Georgina S. Butler[2,3#].

From the [1]Department of Biochemistry & Molecular Biology and [2]Centre for Blood Research, University of British Columbia, 2350 Health Sciences Mall, Vancouver, British Columbia, Canada, V6T 1Z3; [3]Department of Oral Biological & Medical Sciences, University of British Columbia, 2199 Wesbrook Mall, Faculty of Dentistry, Vancouver, British Columbia, Canada, V6T 1Z3; [4]Graduate Program in Bioinformatics, University of British Columbia, 100-570 West 7th Avenue, Vancouver, British Columbia, Canada, V5T 4S6; [5]College of Pharmacy and [6]Medicinal Bioconvergance Research Center, Seoul National University, 151-742, Seoul, Republic of Korea.

Running title: *MMPs enhance proinflammatory YRS activity*

[#]Co-senior authors.

*To whom correspondence should be addressed: Christopher M. Overall: University of British Columbia, 2350 Health Sciences Mall, Room 4.401, Vancouver, British Columbia, Canada, V6T 1Z3; chris.overall@ubc.ca; Tel.(604) 822-2958; Fax. (604) 822-7742.

Supporting Information

Supplementary Figure S1. YRS expression and secretion in THP1 cells was constitutive.
Supplementary Figure S2. Grid showing layout of the cytokine protein array used to analyze conditioned media of human peripheral blood mononuclear-derived macrophages following stimulation by YRS.
Supplementary Figure S3. Validation of TLR-blocking antibody.
Supplementary Figure S4. Full immunoblots.
Supplementary Figure S5. Full Coomassie-stained gels.
Supplementary Figure S6. Full Coomassie-stained PVDF membranes from which bands were microsequenced by Edman degradation shown in Fig. 6 *B*.

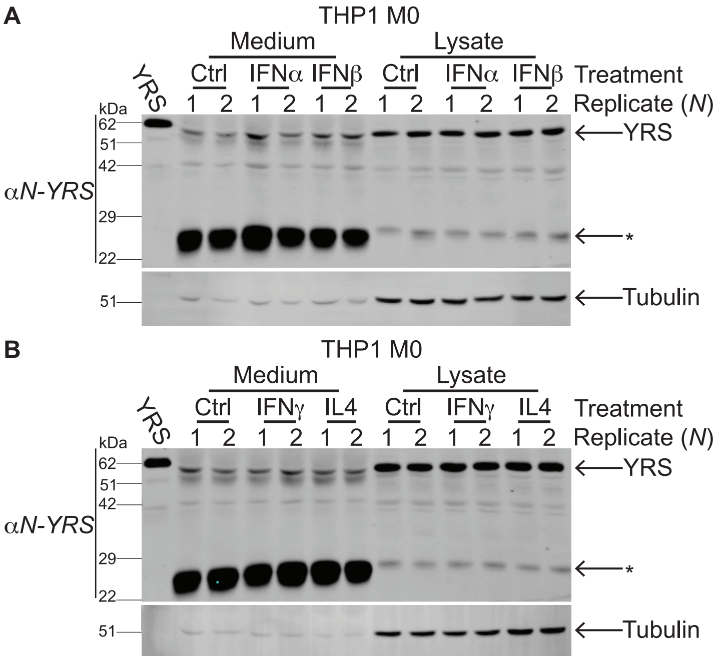

Supplementary Figure S1. YRS expression and secretion in THP1 cells was constitutive. Immunoblots of replicate samples (1 and 2) of PMA-differentiated THP1-derived macrophages (THP1 M0) treated for 48 h with buffer (Ctrl), (*A*) IFNα, IFNβ (100 ng/mL each), or (*B*) IFNγ (20 ng/mL), or IL4 (40 ng/mL). An antibody specific to the N-terminus of YRS (α*N-YRS*) was used to identify YRS in conditioned media and cell lysates. *, nonspecific immunoreactive band. Standards: YRS: Recombinant human YRS (band ~62-kDa); tubulin, loading control. Representative immunoblots after 10% SDS-PAGE are shown of *N* = 2 independent experiments. Uncropped immunoblots are shown in Supplementary Fig. S4 *A* and *B*.

	A	B	C	D	E
1	+				+
2	MIF	IL13	IL1ra	CXCL11	CCL1
3	PAI-1	IL16	IL2	CXCL12	MCP-1
4	TNFα	IL17A	IL4	G-CSF	MIP-1α/β
5	TREM-1	IL17E	IL5	GM-CSF	CCL5
6		IL18	IL6	ICAM-1	TNFSF5
7		IL21	IL8	IFNγ	C5/C5a
8		IL27	IL10	IL1α	CXCL1
9		IL32α	IL12p70	IL1β	IP-10
10	-				+

Supplementary Figure S2. Grid showing layout of the cytokine protein array used to analyze conditioned media of human peripheral blood mononuclear-derived macrophages following stimulation by YRS. The cytokines and chemokines with significant changes in expression in Fig. 1 *C* are boxed. +, positive control spots; –, negative control protein spots.

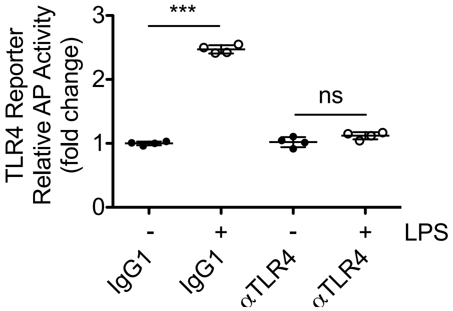

Supplementary Figure S3. Validation of TLR-blocking antibody. HEK293 cells expressing TLR4 with a NF-κB alkaline phosphatase (AP) reporter system were pre-treated for 1 h with 5 μg/mL TLR4-blocking antibody (αTLR4) or isotype control IgG1 prior to treatment ± 100 ng/mL LPS for 18 h. The relative activity of alkaline phosphatase was plotted as fold changes compared to the IgG1 – LPS condition (means ± SD, n = 4) of N = 2 independent experiments. Statistical significance was determined between the – LPS and + LPS conditions using an unpaired two-tailed Student's t-test. *** $p < 0.001$; ns, not significant. Error bars represent SD.

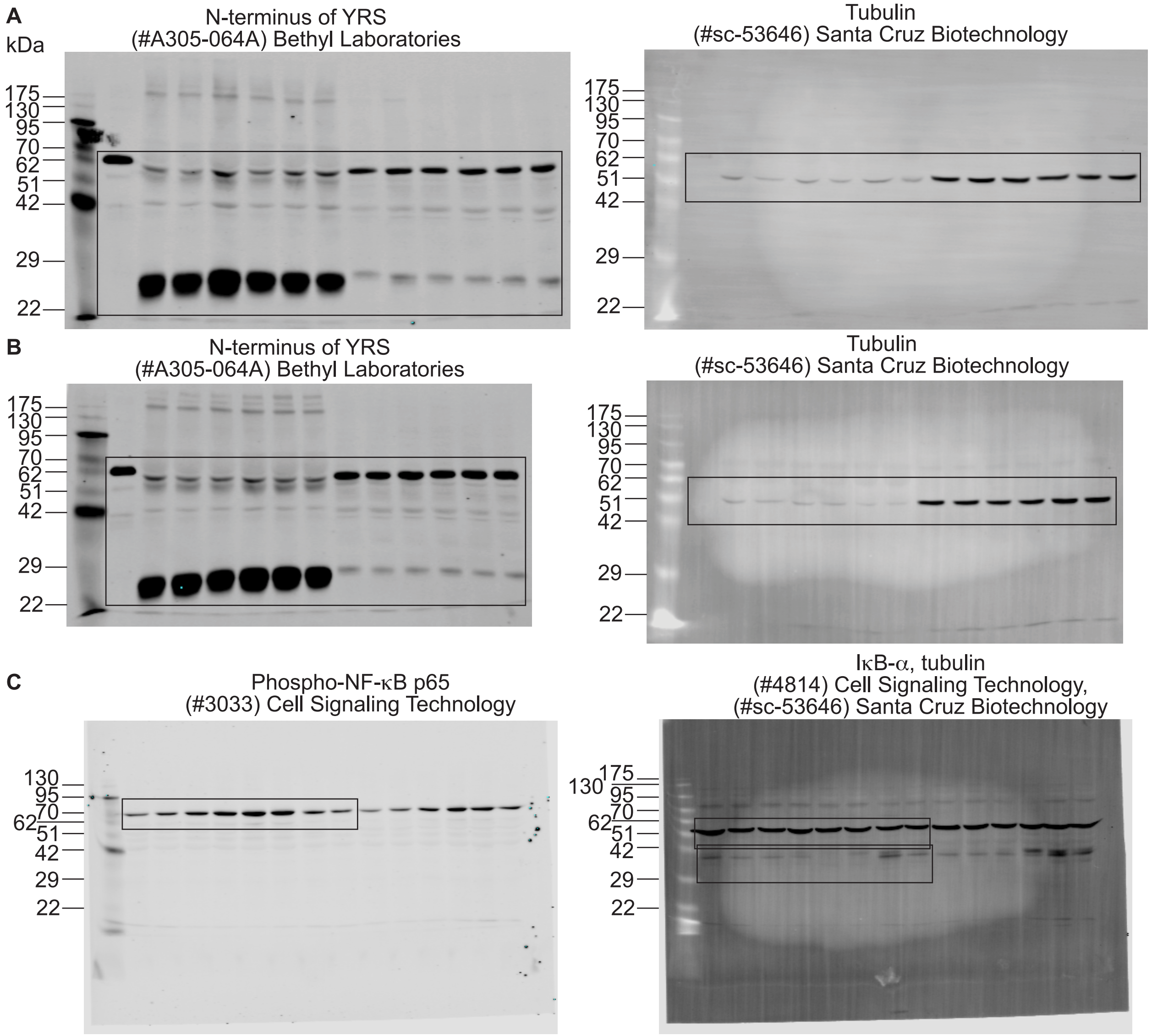

Supplementary Figure S4. Full immunoblots. (*A*) Supplementary Fig. S1 *A*. (*B*) Supplementary Fig. S1 *B*. (*C*) Fig. 2 *A* Immunoblot sections presented in the respective figures are boxed with the details of the antibodies used. kDa, MW of marker proteins are as shown.

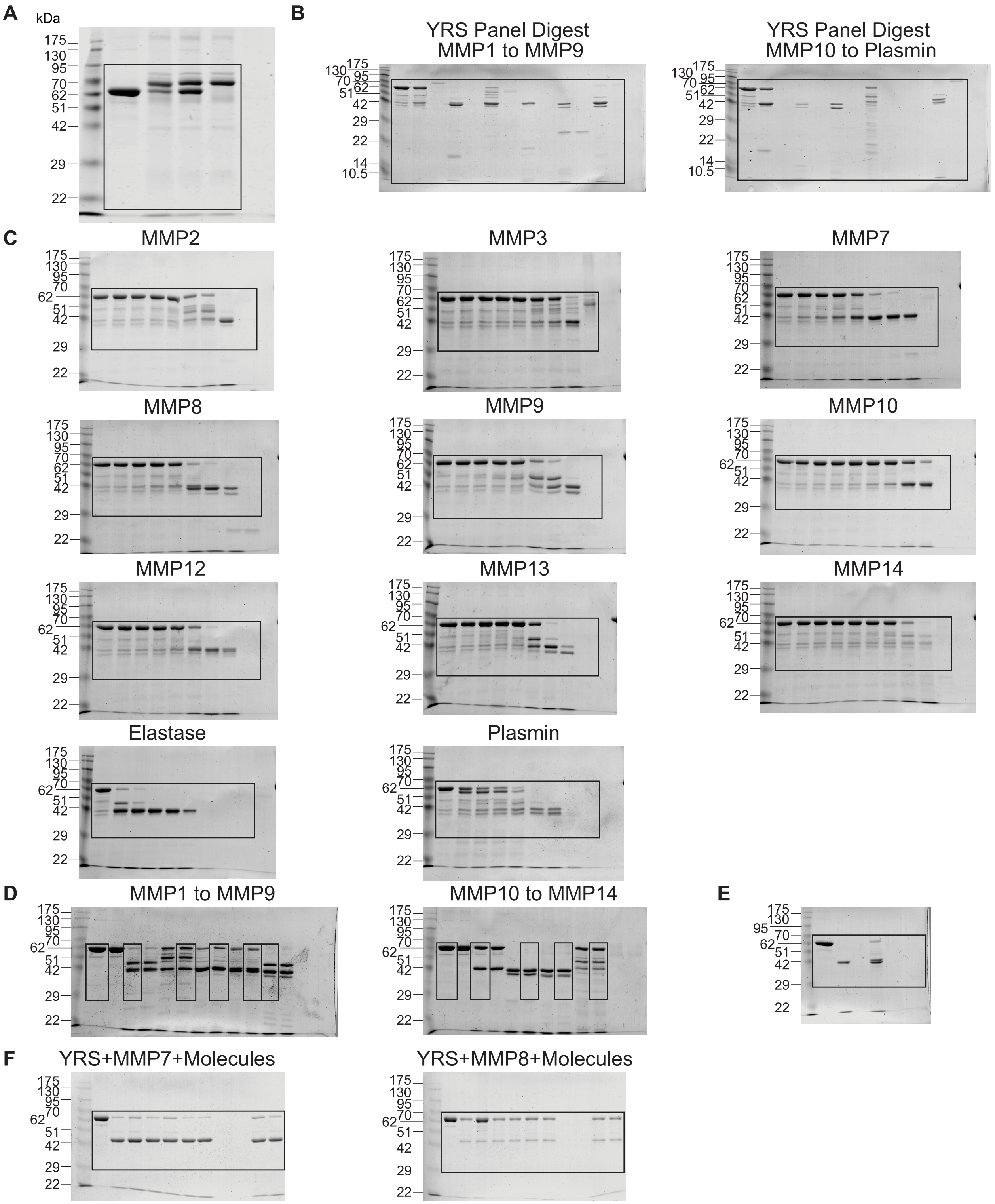

Supplementary Figure S5. Full Coomassie-stained gels. (*A*) Fig. 4 *B*. (*B*) Fig. 5 *A*. (*C*) Fig. 5 *B*. (*D*) Fig. 6 *A*. (*E*) Fig. 9 *A*. (*F*) Fig. 10 *A* and *B*. Gel sections presented in the respective figures are boxed. kDa, MW of marker proteins are as shown.

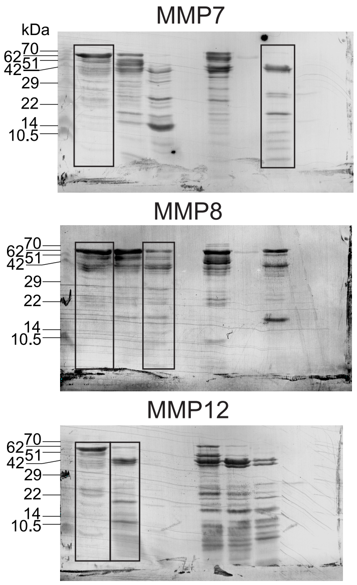

Supplementary Figure S6. Full Coomassie-stained PVDF membranes from which bands were microsequenced by Edman degradation shown in Fig. 6 *B*. Sections presented in the respective figure are boxed. kDa, MW of marker proteins are as shown.
